# NIK regulates MT1-MMP activity and promotes glioma cell invasion independently of the canonical NF-κB pathway

**DOI:** 10.1038/oncsis.2016.39

**Published:** 2016-06-06

**Authors:** C L Duran, D W Lee, J-U Jung, S Ravi, C B Pogue, L G Toussaint, K J Bayless, R Sitcheran

**Affiliations:** 1 Department of Molecular and Cellular Medicine, Texas A&M Health Science Center, College Station, TX, USA; 2 Interdisciplinary Program in Genetics, Texas A&M University, College Station, TX, USA; 3 Medical Sciences Graduate Program, Texas A&M Health Science Center, College Station, TX, USA; 4 Department of Neuroscience and Experimental Therapeutics, Texas A&M Health Science Center, College Station, TX, USA; 5 The Texas Brain and Spine Institute, Bryan, TX, USA

## Abstract

A growing body of evidence implicates the noncanonical NF-κB pathway as a key driver of glioma invasiveness and a major factor underlying poor patient prognoses. Here, we show that NF-κB-inducing kinase (NIK/MAP3K14), a critical upstream regulator of the noncanonical NF-κB pathway, is both necessary and sufficient for cell-intrinsic invasion, as well as invasion induced by the cytokine TWEAK, which is strongly associated with tumor pathogenicity. NIK promotes dramatic alterations in glioma cell morphology that are characterized by extensive membrane branching and elongated pseudopodial protrusions. Correspondingly, NIK increases the phosphorylation, enzymatic activity and pseudopodial localization of membrane type-1 matrix metalloproteinase (MT1-MMP/MMP14), which is associated with enhanced tumor cell invasion of three-dimensional collagen matrices. Moreover, NIK regulates MT1-MMP activity in cells lacking the canonical NF-κB p65 and cRel proteins. Finally, increased expression of NIK is associated with elevated MT1-MMP phosphorylation in orthotopic xenografts and co-expression of NIK and MT1-MMP in human tumors is associated with poor glioma patient survival. These data reveal a novel role of NIK to enhance pseudopodia formation, MT1-MMP enzymatic activity and tumor cell invasion independently of p65. Collectively, our findings underscore the therapeutic potential of approaches targeting NIK in highly invasive tumors.

## Introduction

The persistent invasiveness of high-grade glioma cells into healthy brain tissue is a major factor underlying the therapy resistance and poor prognosis of this malignancy. NF-κB transcription factors have been shown to have key roles in regulating tumor-promoting functions including cell migration and invasion.^1, 2^ There are two main pathways controlling NF-κB activation. In the context of glioma, most studies have focused on the canonical NF-κB pathway, which is dependent on IκB kinase-β (IKKβ) and mediated by p65 (RelA)- or cRel-containing transcription factor complexes. However, we and others have demonstrated that constitutive, noncanonical NF-κB signaling, mediated by RelB-p52 complexes, predominates in aggressive, mesenchymal glioma where it promotes cell migration, invasion and tumor recurrence.^3, 4, 5, 6^ Activation of the noncanonical NF-κB pathway is dependent on NF-κB-inducing kinase (NIK, also known as MAP3K14), a constitutively active kinase that is continuously targeted for proteasomal degradation in unstimulated cells.^7, 8, 9^ In response to specific cytokines, NIK degradation is attenuated, resulting in the activation of IKKα, phosphorylation-dependent proteolytic processing of the RelB-inhibitory protein p100 to p52 and nuclear translocation of RelB-p52 heterodimers.^10^ We have recently shown that TNF-like weak inducer of apoptosis (TWEAK, also known as TNFSF12) preferentially activates noncanonical NF-κB RelB and p52 proteins and promotes the invasive properties of glioma cells.^3^

Tumors must degrade the surrounding extracellular matrix (ECM) to invade into nearby healthy tissue.^11^ Invasive cancer cell phenotypes show elevated formation of invadopodia, which are specialized actin- and cortactin-rich membrane protrusions that mediate attachment to and degradation of the ECM.^12^ Invadopodia formation in two-dimensions (2D) is associated with greater invasive behavior in three-dimensions (3D), where cells must extend larger protrusions, termed pseudopodia, to migrate successfully.^13, 14^ Membrane type-1 matrix metalloproteinase (MT1-MMP, also known as MMP14) is the predominant ECM-degrading enzyme localized to invadopodia.^11, 13, 14^ MT1-MMP is highly expressed in invasive human cancers and is associated with poor patient survival.^15, 16, 17, 18^ MT1-MMP is activated by furin cleavage,^19, 20^ and once phosphorylated on Y573,^21^ MT1-MMP is directed to the plasma membrane,^22^ where it can degrade multiple ECM proteins.^23^ Notably, the signals that regulate MT1-MMP localization to the cell surface during invasion are not fully understood.

Several studies have established a role for canonical NF-κB-dependent (p65-mediated) regulation of MT1-MMP expression.^24, 25^ In addition, Fritz and Radziwill^26^ demonstrated that noncanonical NF-κB signaling (RelB-p52-mediated) regulates MT1-MMP expression and tumor cell invasion induced by the scaffold protein CNK1. Thus, although both canonical and noncanonical NF-κB signaling have been linked to regulating MT1-MMP expression, whether these pathways control activation and localization of MT1-MMP during invasion have not been established. Importantly, the role of NIK in both constitutive and TWEAK-induced invasion of glioma cells is not well understood. In this study, we establish novel functions for NIK in regulating MT1-MMP.

## Results

### NIK is required for constitutive and TWEAK-induced glioma cell invasion

**Figure 1:**
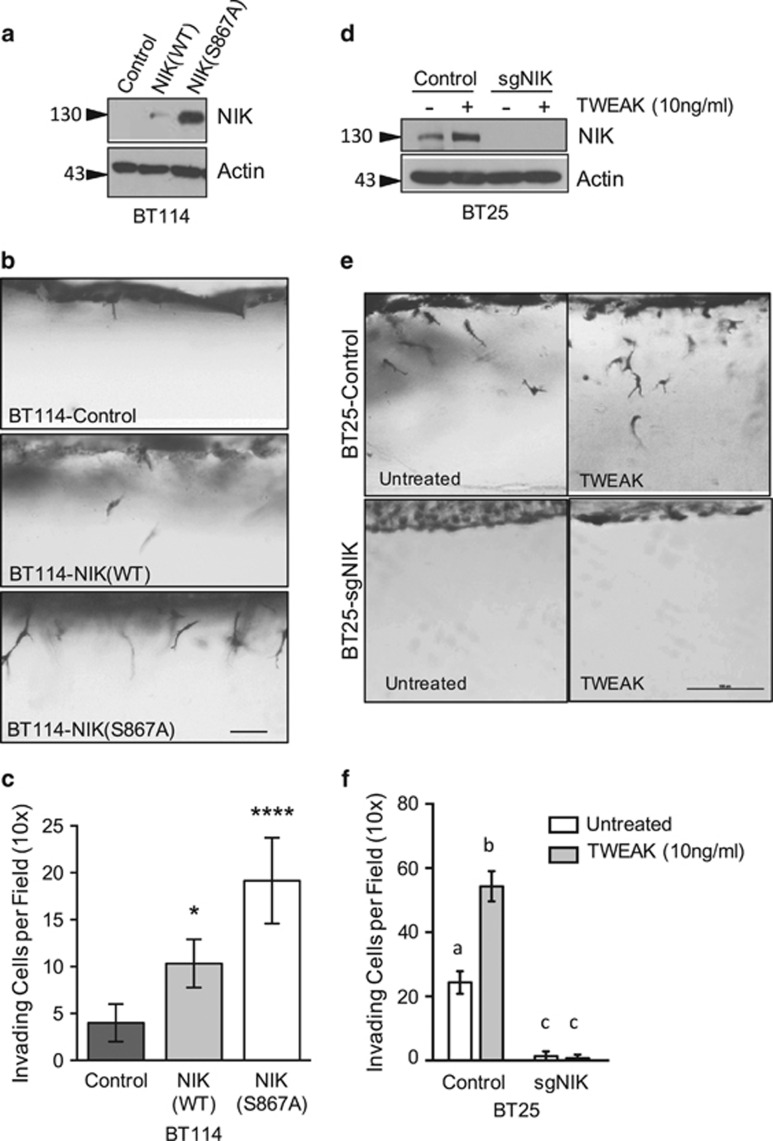
NIK promotes glioma cell invasion. (**a**) Western blot analysis of BT114 glioma cells transduced with lentiviral vectors expressing luciferase (Control), NIK(WT) or NIK(S867A). Whole-cell detergent lysates were probed with indicated antibodies. (**b**) Side view images of BT114-Control, -NIK(WT) and -NIK(S867A) cells after 24 h invasion in 3D collagen matrices. Cells in collagen matrices were fixed, stained with toluidine blue, cut into thin slices and imaged with light microscopy. Scale bar, 50 μm. (**c**) Quantification of invasion density of BT114-Control, -NIK(WT) and -NIK(S867A) cells in (**b**) represented as the average numbers of invading cells per 1-mm^2^ field (top-view)±s.d. after 24 h of invasion. At least six wells were quantified per experiment for four independent experiments. Graph shows mean±s.d. for a representative experiment. Statistical significance was calculated with two-way analysis of variance (ANOVA) and Tukey's HSD post test. Multiplicity-adjusted *P*-values: *0.0109 for Control vs NIK(WT) and ****<0.0001 for Control vs NIK(S876A). (**d**) Western blot analysis of whole-cell lysates from BT25-Control and clonally selected BT25-sgNIK cells. Cells were first treated with MG132 for 1 h to prevent NIK degradation and then untreated or treated with TWEAK (10 ng/ml), as indicated for 5 h. Extracts were probed with indicated antibodies. (**e**) Side view images of invasion assays with untreated or TWEAK-treated (10 ng/ml) BT25-Control and BT25-sgNIK cells allowed to invade 3D collagen matrices for 24 h. Scale bar=100 μm. (**f**) Invasion densities for (**e**) were quantified as described above in (**c**). At least three wells of invading cells were quantified per experiment for three independent experiments. Graph shows mean±s.d. from a representative experiment. Different letters represent statistically significant differences calculated with two-way ANOVA and Tukey's HSD post test. For all differences (a vs b/c, b vs a/c and c vs a/b), multiplicity-adjusted *P*-values are all <0.0001.

We previously demonstrated that patient-derived glioma cell lines exhibit distinct invasive potentials that correlate more strongly with noncanonical NF-κB/RelB signaling than with canonical NF-κB/p65 activity.^3, 4^ To address the role of NIK, a key upstream regulator of noncanonical NF-κB signaling, in glioma invasion and pathogenesis, we first sought to determine whether NIK was sufficient to promote cell invasion in BT114 glioma cells, which exhibit low invasive activity.^3^ In addition to expressing wild-type NIK (NIK(WT)), which is continuously degraded under unstimulated conditions, we also used a more stable form of NIK that allowed greater protein accumulation and facile immunological detection. Specifically, a S867A substitution at the conserved TBK1 phosphorylation site renders human NIK resistant to degradation,^8^ and immunoblot analysis of BT114 glioma cells confirmed that NIK(S867A) is expressed at higher levels than NIK(WT) (Figure 1a). Using 3D collagen type I invasion assays, we observed that NIK-transfected cells were more invasive than controls cells, and NIK(S867A) exerted a significantly stronger effect than NIK(WT) (Figures 1b and c). Furthermore, ectopic expression of NIK in several additional glioma lines, including BT116, U87 and BT25 cells, promoted cell invasion in this assay (Supplementary Figure 1).

Next, we investigated the effects of NIK knockdown using CRISPR/Cas9/small-guide (sg)RNA-mediated deletion of *NIK.* For these experiments, we took advantage of the highly invasive BT25 glioma line.^3^ NIK protein was no longer detectable in both untreated and TWEAK-treated BT25-sgNIK cells, indicating efficient sgRNA-mediated deletion of *NIK* (Figure 1d). We also confirmed the loss of NIK expression in BT25-sgNIK cells by immunofluorescence staining, flow cytometry and impaired p100 processing and p52 nuclear localization (Supplementary Figures 2A-C). Importantly, loss of NIK significantly diminished the invasive potential of both untreated, as well as TWEAK-treated, BT25-sgNIK cells (Figures 1e and f). Similarly, loss of NIK in BT114-sgNIK cells significantly attenuated invasion in unstimulated and TWEAK-treated cells (Supplementary Figures 2D-F). Together, these data identify a critical role for NIK in regulating both constitutive and TWEAK-induced glioma cell invasion.

### NIK promotes pseudopodia formation

**Figure 2:**
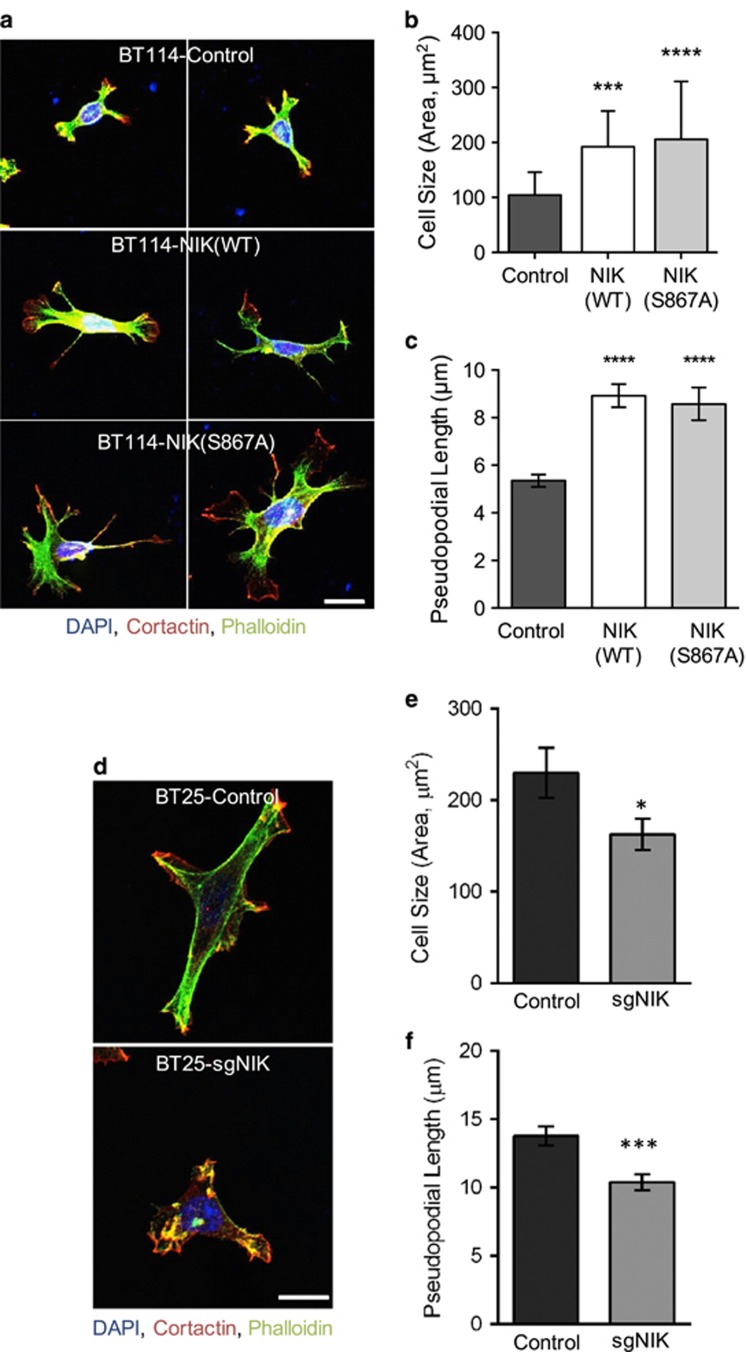
NIK enhances pseudopodia formation. (**a**) BT114 cells expressing Luciferase (Control), NIK(WT) or NIK(S867A) were seeded onto collagen-coated coverslips and fixed after 16 h. Cells were stained with DAPI (blue), Alexa Fluor 488-phalloidin (green) and an antibody specific for cortactin (red) and imaged using confocal microscopy. Images of representative cells are shown. Scale bar=10 μm. (**b**, **c**) Quantification of cell size and pseudopodial length of BT114 cells in (**a**). At least 65 cells (Control, NIK(WT) or NIK(S867A)) from three independent staining experiments were used for the following blinded quantifications: (**b**) average cell size (area, μm^2^)±s.e.m. Statistical significance was calculated with one-way analysis of variance (ANOVA) and Tukey's HSD post test. Multiplicity-adjusted *P*-values: ***0.0002 for Control vs NIK(WT); ****<0.0001 for Control vs NIK(S867A), (**c**) average length (μm) of pseudopodial structures extended from the cell body±s.e.m. Statistical significance was calculated with one-way ANOVA and Tukey's HSD post test. Multiplicity-adjusted *P*-values: ****<0.0001 for Control vs NIK(WT) and for Control vs NIK(S867A). (**d**) BT25-Control or BT25-sgNIK cells were treated and imaged as described in (**a**). Representative cells shown. Scale bar=10 μm. (**e**) Average cell size (area, μm^2^)±s.e.m. for BT25-Control or BT25-sgNIK cells. Unpaired *t*-test *P*-value: *0.0330 for Control vs sgNIK. (**f**) Average length (μm) of pseudopodial structures extended from the cell body±s.e.m. Unpaired *t*-test *P*-value: ***<0.0002 for Control vs sgNIK. At least 50 cells from three independent experiments were used for quantification (**e**, **f**).

Because changes in morphology can be predictive of cell motility and metastatic potential,^27^ we sought to determine whether the ability of NIK to promote invasion of glioma cells was associated with changes in cell shape. Examination of BT114 cells in 2D culture revealed that both NIK(WT) and NIK(S867A) significantly altered cell morphology, specifically by inducing the formation of F-actin- and cortactin-dense, branched cellular processes resembling the pseudopodia of invasive cells (Figure 2a). Quantification of these features (Supplementary 3A) revealed that total cell area as well as the length of extended pseudopodial cell processes were increased in both NIK(WT) and NIK(S867A) cells, compared with vector control cells (Figures 2b and c). Conversely, we observed that BT25-sgNIK cells were smaller and less elongated compared with BT25-control cells (Figures 2d and f). These data establish a role for NIK in increasing the formation of pseudopodial membrane protrusions, cell size and cell elongation.

### NIK increases MT1-MMP pseudopodial localization and enzymatic activity

**Figure 3:**
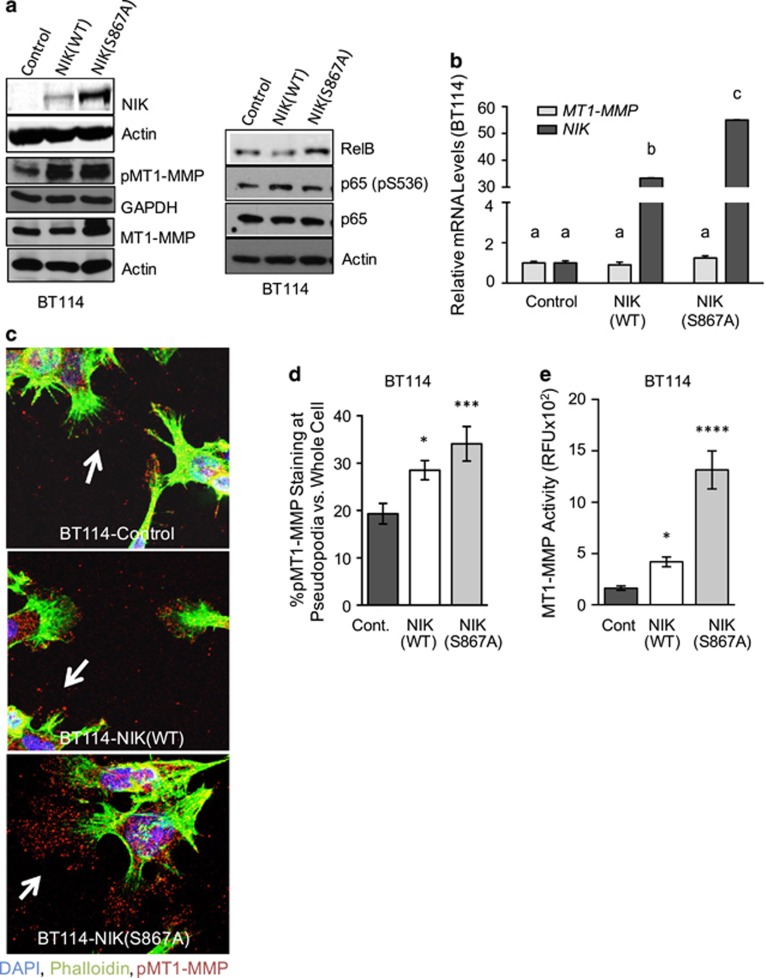
NIK increases MT1-MMP activity and pseudopodial localization. (**a**) Representative western blot analysis (*n=*4) of BT114-Control, -NIK(WT) or -NIK(S867A) cells. Whole-cell lysates were probed with indicated antibodies. (**b**) qPCR analysis was performed to analyze expression of *MT1-MMP* and *NIK* in BT114 cells expressing Control, NIK(WT) or NIK(S867A). Graph shows fold-change *MT1-MMP* and *NIK* expression relative to BT114-Control cells. Average gene expression was calculated from triplicate wells from a representative experiment that was repeated three times. *GAPDH* expression was used as the endogenous control. Statistical analysis of gene expression data was calculated using two-way analysis of variance (ANOVA) with Tukey's HSD post test. Different letters indicate statistically significant differences with multiplicity-adjusted *P*-values<0.0001 for all comparisons. (**c**) BT114 cells expressing Control, NIK(WT) or NIK(S867A) grown on collagen-coated coverslips were stained with DAPI (blue), Alexa Fluor 488-phalloidin (green) and anti-pMT1-MMP (Y573) (red). Confocal microscopy was used to evaluate subcellular localization of pMT1-MMP. Images of representative cells from one of six experiments are shown. Arrows indicate pMT1-MMP localized to pseudopodia. (**d**) Quantification of pMT1-MMP staining that localized to pseudopodia was performed using images obtained in (**c**) and normalized to total cellular pMT1-MMP staining: at least 30 cells from six different experiments were used for quantification. One-way ANOVA with Tukey's HSD post test multiplicity-adjusted *P*-values: *0.0394 Control vs NIK(WT); ***0.0009 Control vs NIK(S867A). (**e**) MT1-MMP activity was assayed in the indicated cells after 16 h of invasion using the Sensolyte 520-MMP14 Assay kit (AnaSpec) with modifications to specifically quantify MT1-MMP activity.^54^ Representative graph from three experiments depicts average RFU (relative fluorescence units)±s.d. One-way ANOVA with Holm-Sidak post test multiplicity-adjusted *P*-values: *0.0301 for NIK(WT) vs Control; ****<0.0001 for NIK(S867A) vs Control.

The matrix-degrading enzyme, MT1-MMP (also known as MMP14), is the predominant proteolytic component of invasive pseudopodia.^11, 13, 14^ Unlike soluble, secreted MMPs, cell surface localization of active, phosphorylated MT1-MMP (pMT1-MMP, Y573) is required for degradation of ECM proteins and tumor cell invasion.^21, 22, 28, 29, 30^ We therefore investigated whether NIK promoted pseudopodia formation through regulation of MT1-MMP. First, we noted a positive correlation between glioma invasion, NIK expression, MT1-MMP expression and MT1-MMP enzymatic activity in BT25 and BT114 cells (Supplementary Figure 4). We previously demonstrated that MT1-MMP mRNA levels were not altered by TWEAK treatment.^3^ Consistent with this observation, we found that NIK(WT) did not significantly affect the expression of MT1-MMP at protein or mRNA level in BT114 cells (Figures 3a and b). MT1-MMP mRNA levels were unaltered in BT114-sgNIK cells irrespective of treatment with TWEAK (Supplementary Figure 2G). The increase of MT1-MMP in BT114-NIK(S867A) cells compared with BT114-NIK(WT) cells is likely due to higher expression levels of transfected NIK in the former compared with the latter cells (Figures 3a and b). BT114-NIK(S867A) cells also showed slightly increased levels of RelB, but total and phosphorylated (P536) p65 levels remain unchanged. However, BT114 cells overexpressing NIK(WT) and NIK(S867A) exhibited increased levels of pMT1-MMP (Figure 3a), suggesting that NIK may regulate MT1-MMP intracellular localization.

**Figure 4:**
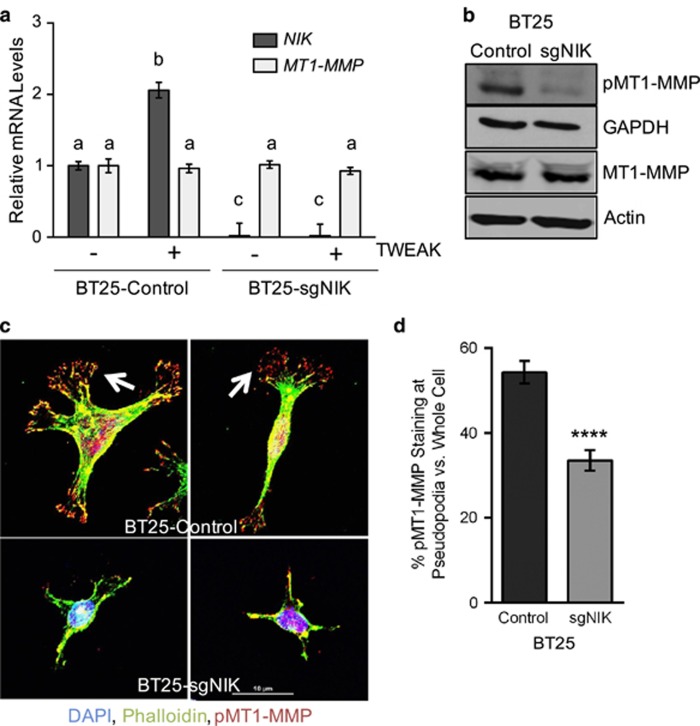
Loss of NIK decreases invasion and results in loss of pseuodopodial pMT1-MMP localization. (**a**) qPCR analysis of *NIK* and *MT1-MMP* mRNA expression in BT25-Control and BT25-sgNIK cells that were untreated or treated with TWEAK for 24 h. Graph shows data from triplicate wells from one representative experiment that was repeated three times. *GAPDH* expression was used as endogenous control. Statistical analysis of gene expression data was calculated using two-way analysis of variance (ANOVA) with Tukey's HSD post test. Different letters indicate statistically significant differences with the following multiplicity-adjusted *P*-values: *P<*0.0001 for ‘a vs b' *P=*0.001 for ‘a vs c'. (**b**) Representative western blot analysis of pMT1-MMP (Y573) and MT1-MMP expression in BT25-Control and BT25-sgNIK cells. Whole-cell lysates were probed with the indicated antibodies. (**c**) BT25-Control and BT25-sgNIK cells grown on collagen-coated coverslips were stained with DAPI (blue), Alexa Fluor 488-phalloidin (green) and anti-pMT1-MMP (Y573) (red). Confocal microscopy was used to evaluate subcellular localization of pMT1-MMP. Images of representative cells from one of three experiments are shown. Arrows indicate pMT1-MMP localized to pseudopodia. Scale bar=10 μm. (**d**) Quantification of pMT1-MMP staining that localized to pseudopodia was performed using images obtained in (**c**) and normalized to total cellular pMT1-MMP staining: BT25-Control (*n=* 30 cells) and BT25-sgNIK (*n=*30 cells). Cells from three different experiments were used for quantification. Unpaired Student's test *P*-value ****<0.0001.

We next evaluated the effect of ectopic NIK expression on pMT1-MMP subcellular distribution. BT114-Control cells exhibited a punctate pMT1-MMP staining pattern throughout pseudopodial structures at the cell periphery (Figure 3c). When compared with these controls, BT114 cells expressing NIK(WT) or NIK(S867A) showed an increase in pMT1-MMP pseudopodial staining, with NIK(S867A) having a stronger effect than NIK(WT) (Figure 3c). Quantification of pMT1-MMP staining demonstrated that the percent of pMT1-MMP at the pseudopodia was significantly increased in NIK(WT)- and NIK(S867A)-expressing cells, and correlated with levels of NIK expression (Figure 3d and Supplementary Figure 3). Volumetric views of these immunostaining experiments demonstrated that pMT1-MMP co-localized with the pseudopodia marker cortactin (Supplementary Figure 3B), which is required for pseudopodia assembly and maintenance.^12^ Consistent with these observations, we observed that MT1-MMP enzymatic activity was increased in cells expressing NIK(WT) or NIK(S867A) (Figure 3e). Loss of NIK in BT25-sgNIK cells did not affect the expression of MT1-MMP mRNA or total protein, but did diminish levels of pMT1-MMP (Figures 4a and b). Furthermore, we also observed that pseudopodial localization of pMT1-MMP was significantly diminished in the absence of NIK (Figures 4c and d). Taken together, both gain-of-function and loss-of-function studies support a working model whereby NIK predominantly regulates MT1-MMP at the post-transcriptional level.

To test the hypothesis that NIK enhances MT1-MMP activity post-transcriptionally, we used a heterologous assay involving ectopic co-expression of NIK(S867A) and MT1-MMP in HEK293FT cells. Control HEK293FT cells (vector only) lack MT1-MMP, exhibit low MT1-MMP activity and do not invade collagen (Supplementary Figures 5A,B,D and E). Ectopic expression of NIK(S867A) alone in HEK293FT cells did not increase endogenous MT1-MMP protein, mRNA or enzymatic activity (Supplementary Figures 5A,B and C), and did not enhance the invasive potential of these cells (Supplementary Figures 5D and E). While ectopic expression of a CMV-driven MT1-MMP promoted HEK293FT cell invasion as previously reported,^31^ co-expression of NIK and CMV-MT1-MMP significantly increased HEK293FT cell invasion (see side view images of collagen matrices and quantification of invasion cell densities; Supplementary Figures 5D and E). Consistent with increased cell invasion, we observed that HEK293FT cells co-expressing NIK and MT1-MMP exhibited increased MT1-MMP enzymatic activity compared with expression of MT1-MMP alone (Supplementary Figure 5B). These data demonstrate that NIK can enhance MT1-MMP enzymatic activity and localization within pseudopodia without increasing MT1-MMP expression.

### TWEAK and NIK promote invasion and increase pseudopodial pMT1-MMP localization independently of the canonical NF-κB pathway

NIK is required for activation of noncanonical NF-κB transcription factors, but can also regulate canonical NF-κB proteins (p65 and cRel).^32, 33, 34^ As RelA/p65 was previously reported to regulate expression of MT1-MMP,^24, 25^ we sought to determine whether TWEAK- or NIK-induced invasion and regulation of pMT1-MMP required canonical NF-κB signaling. We first noted that neither NIK(WT) or NIK(S867A) affected p65 phosphorylation (pS536), a marker of p65 transcriptional activation^35^ (see Figure 3a). Moreover, neither TWEAK treatment nor NIK expression significantly affected p65 nuclear translocation^3^ (Supplementary Figure 6), suggesting that NIK does not significantly enhance p65 activity in these cells.

**Figure 5:**
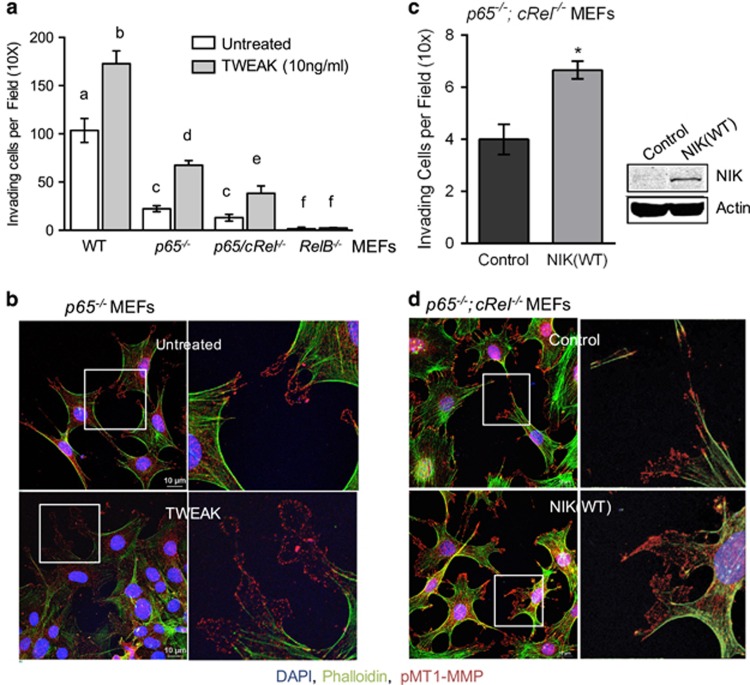
TWEAK increases invasion and pMT1-MMP pseudopodia localization in the absence of canonical NF-κB p65/cRel proteins. (**a**) WT MEFs and NF-κB-null MEFs were allowed to invade collagen matrices±10 ng/ml TWEAK. WT represents pooled invasion data from *p65*^*+/+*^, *p65/cRel*^*+/+*^ and *RelB*^*+/+*^ MEFs with a total of *n=*9 per treatment (±TWEAK). Three independent experiments were performed counting three invading wells per treatment. Graph depicts one representative experiment. Differences between untreated and TWEAK-treated samples of a given genotype, as well as differences among untreated or TWEAK-treated cells of different genotypes were analyzed using two-way analysis of variance (ANOVA) with Tukey's HSD post test. Statistically significant differences are indicated by different letters. Multiplicity-adjusted *P*-values: ****<0.0001 between all letters, except ***<0.001 for ‘c vs e' and *<0.01 for ‘c vs f.' (**b**) *p65*^*−/−*^ MEFs were seeded onto collagen-coated coverslips and treated with or without (untreated) 10 ng/ml TWEAK for 4 h. Cells were fixed and stained with anti-pMT1-MMP (red), Alexa Fluor 488-phalloidin (green) and DAPI (blue), and imaged using confocal microscopy. Scale bar=10 μm. Right panels show the zoomed portion of the boxed image on the left. Images of representative cells from one of three experiments are shown. (**c**) Invasion density of *p65*^*−/−*^*;cRel*^*−/−*^ knockout MEFs transduced to express vector (Control) or NIK(WT). At least four wells were quantified per treatment for three independent experiments. Graph depicts one representative experiment. Unpaired *t*-test *P*-value: *0.01. Panel shows western blot analysis of *p65*^*−/−*^*; cRel*^*−/−*^ knockout MEFs expressing vector (Control) or NIK(WT) confirming exogenous NIK expression. (**d**) *p65*^*−/−*^; *cRel*^*−/−*^MEFs were seeded onto collagen-coated coverslips, and treated and immunostained as described in (**b**). Scale bar, 10 μm.

To test the effects of TWEAK and NIK on invasion in the absence of specific NF-κB proteins (Supplementary Figures 7A-C), we exploited the ability of mouse embryonic fibroblasts (MEFs) to invade collagen in an MMP-dependent manner (Supplementary Figure 7D). MEFs derived from homozygous *p65*^*−/−*^ or *p65*^*−/−*^*;cRel*^*−/−*^ mice showed significantly reduced invasion compared with their corresponding pooled wild-type MEFs, while *RelB*^*−/−*^MEF invasion was almost completely abrogated (Figure 5a). TWEAK treatment significantly enhanced invasion in all MEFs, with the exception of *RelB*^*−/−*^ MEFs (Figure 5a and Supplementary Figure 7E). Notably, TWEAK-enhanced invasion of *p65*^*−/−*^ MEFs was accompanied by a significant increase of pMT1-MMP located within pseudopodia (Figure 5b and Supplementary Figure 7F).

We explored the invasion potential of *NIK*^*−/−*^ MEFs and their corresponding *NIK*^*+/+*^ wild-type controls.^36^ In contrast to the wild-type control MEFs of NF-κB mutant strains (see above), *NIK*^*+/+*^ MEFs were only minimally invasive in 3D collagen matrices, as were *NIK*^*−/−*^ MEFs (Supplementary Figure 8A). We speculate that the variability in WT MEF invasion may be due to different genetic backgrounds of mice from which the MEFs were isolated. Regardless, the *NIK*^*+/+*^ MEFs exhibited robust TWEAK-induced invasion, which was significantly impaired in *NIK*^*−/−*^ MEFs (Supplementary Figure 8A). Moreover, we also observed decreased pseudopodial localization of pMT1-MMP in *NIK*^*−/−*^ MEFs compared with *NIK*^*+/+*^ MEFs (Supplementary Figures 8B and C).

Next, we tested whether NIK is sufficient to promote MEF invasion in the absence of canonical NF-κB proteins. Expression of NIK(WT) in *p65*^*−/−*^*;cRel*^*−/−*^ MEFs increased invasion (Figure 5c) and pseudopodial localization of pMT1-MMP (Figure 5d). Moreover, expression of NIK(WT) did not significantly alter expression of MT1-MMP (Supplementary Figure 7G), consistent with our results in BT114 cells (Figure 3b). Together, these results demonstrate that TWEAK and NIK promote invasion and pMT1-MMP localization within pseudopodia, independently of the canonical NF-κB pathway.

### NIK expression is associated with increased pMT1-MMP in orthotopic xenograft tumors

**Figure 6:**
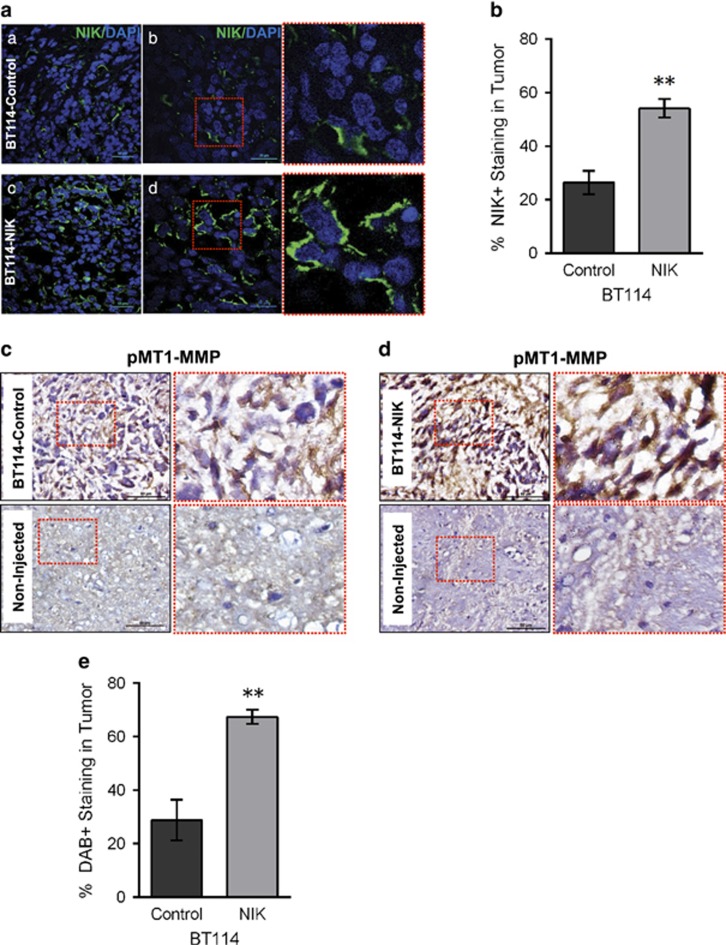
NIK increases tumor growth and pMT1-MMP *in vivo*. (**a**) Sections from BT114-Control or BT114-NIK xenograft tumors^3^ were stained with DAPI (blue) and anti-NIK (green), and imaged using confocal microscopy. Representative images from at least three independent tumors from each group are shown. Scale bars, 20 μm (panels a, c) and 50 μm (panels b, d). Red boxes on sections from b and d are shown magnified on the right. (**b**) Graph shows percent NIK expression in tumors quantified from five sections of each tumor using Image J software analysis. Data are reported as mean±s.e.m. **P=0.0011 vs BT114-Control using unpaired Student's *t*-test. (**c**, **d**) Immunohistochemical diaminobenzidine (DAB) staining (brown) of sections from BT114-Control (**c**) or BT114-NIK (**d**) tumors with anti-pMT1-MMP (Y573) antibody. The portion of the image marked with a red dotted square is shown as a zoomed image on the right. Sections from non-injected brain region are shown as a negative control. Scale bar=50 μm. (**e**) Graph shows quantification of pMT1-MMP staining (**c**, **d**) from five sections of tumors derived from BT114-Control or BT114-NIK cells using IHC profiler^59^ to measure positive pixel counts for DAB staining; data are reported as mean±s.e.m **P=0.0014 vs BT114-Control using unpaired Student's *t*-test.

We previously demonstrated that BT114-NIK cells formed larger tumors *in vivo*, compared with BT114-Control cells.^3^ We used tumor tissue from these animals for immunohistochemical analyses. Immunofluorescence staining verified increased NIK expression in BT114-NIK tumors compared with BT114-Control tumors (Figures 6a and b). Adjacent tumor sections were used for immunohistochemical staining, which revealed increased expression of pMT1-MMP in BT114-NIK tumors, compared with BT114-Control tumors (Figures 6c–e). As a control, we examined pMT1-MMP staining in sections from the non-injected brain hemisphere without tumor growth (Figures 6c and d; bottom panels). These data demonstrate that increased NIK expression in tumors increases levels of pMT1-MMP, which is associated with increased tumor spreading.

### NIK and MT1-MMP expression in human glioma correlates with poor survival

**Figure 7:**
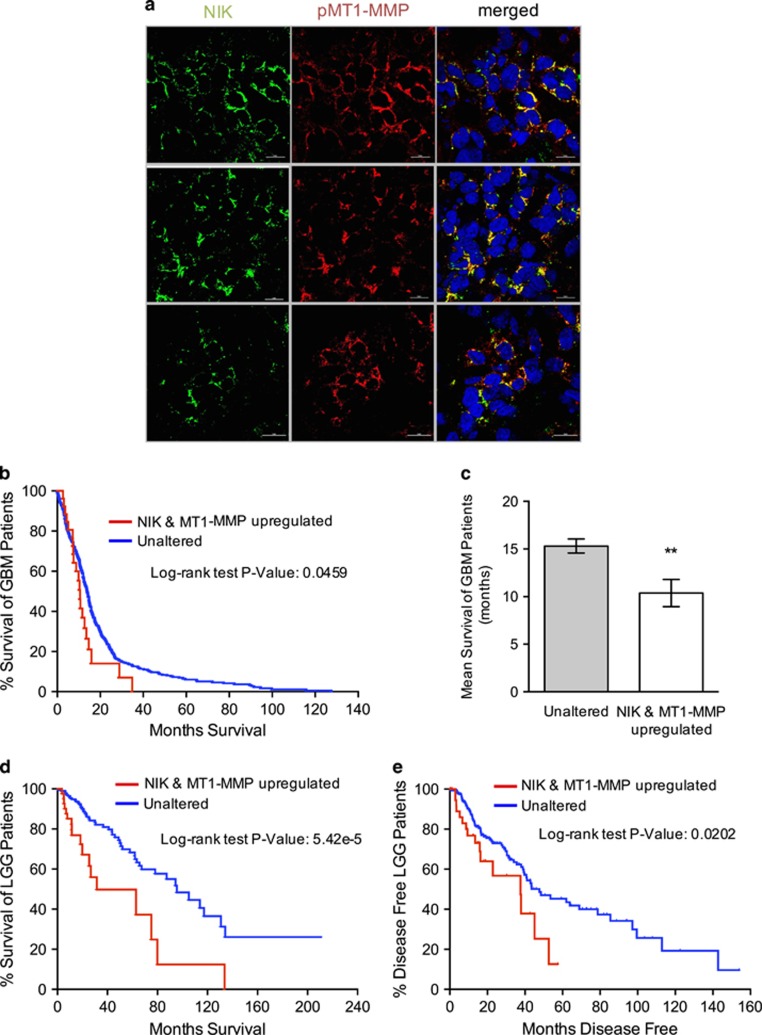
NIK and MT1-MMP are co-expressed in human glioma and correlate with poor patient survival. (**a**) Sections of human glioma *ex vivo* tissue were stained with antibodies specific for pMT1-MMP (Y573) (red) and NIK (pre-conjugated with AF488; green). Representative images are shown from three tumors. (**b**) Kaplan–Meier curve representing TCGA patient survival data from GBM patients with upregulated NIK (MAP3K14) and MT1-MMP (MMP14) mRNA expression. Data were downloaded from cBioPortal for Cancer Genomics TGCA, Cell 2013 data set.^57^ Log-rank Mantel-Cox test indicates statistical significance with *P=*0.0459. (**c**) Mean survival (months) of GBM patients with unaltered (*n=*509) or upregulated NIK and MT1-MMP expression (*n=*27). Unpaired Welch *t*-test indicates statistical significance with **P=0.0038. (**d**) Kaplan–Meier curve representing TCGA patient survival data from LGG patients with upregulated NIK and MT1-MMP mRNA expression (LGG provisional raw data at the NCI). Log-rank Mantel-Cox test indicates statistical significance with *P=*5.42e^*−*5^. (**e**) Kaplan–Meier curve representing disease-free time for LGG patients with upregulated NIK and MT1-MMP. Log-rank Mantel-Cox test indicates statistical significance with *P=*0.0202.

To investigate the significance of NIK and MT1-MMP in human tumors, we examined their expression *ex vivo* in human glioma tissue. Results from these experiments revealed co-expression of NIK and pMT1-MMP in three patient-derived tumor samples (Figure 7a). To determine whether the observed co-expression of NIK and MT1-MMP is relevant for disease pathogenesis, we analyzed NIK and MT1-MMP expression in TCGA data sets through the cBioPortal Cancer Genome for Cancer Genomics.^37, 38^ Kaplan–Meier plots reveal that increased NIK and MT1-MMP mRNA expression correlates with poor survival of glioblastoma (GBM) patients (Figures 7b and c). The mean survival of GBM patients with increased NIK and MT1-MMP expression (5% of cases) is 10.6 months compared with 14.1 months for patients with unaltered expression (Figure 7c). In lower grade glioma (LGG), the correlation between high NIK and MT1-MMP expression (12% of cases) and poor patient survival was even higher (Figure 7d), suggesting that high NIK and MT1-MMP expression is a prognostic indicator for LGGs that are likely to progress to more aggressive tumors. Furthermore, the time to disease progression for LGG patients with high NIK and MT1-MMP expression is significantly shorter and is reflected by a higher rate of relapse (Figure 7e). Collectively, these results demonstrate a strong correlation between NIK and MT1-MMP expression levels and glioma pathogenesis.

## Discussion

Aberrant activation of NIK has been shown to have oncogenic roles in several cancers, including melanoma, ovarian cancer and multiple myeloma,^39, 40, 41^ primarily through regulation of proliferation and cell survival.^33, 41, 42^ However, the role of NIK in CNS tumor pathogenesis, and particularly in tumor cell invasion, has not been clearly established. Here, we demonstrate that NIK has a critical role in regulating MT1-MMP phosphorylation, pseudopodial localization and enzymatic activity to drive cell invasion. Our data suggest that NIK regulates MT1-MMP activity through a mechanism that is both post-transcriptional and indirect. A post-transcriptional process is indicated because we observed that NIK increases MT1-MMP phosphorylation and activity, but does not affect MT1-MMP mRNA expression (Figures 3b and 4a). As phosphorylation of Y573 is the critical step in enhancing movement of MT1-MMP to the cell surface,^22^ and NIK is a serine-threonine kinase, we speculate that NIK-induced MT1-MMP phosphorylation is indirect. However, we cannot rule out the possibility that NIK phosphorylates MT1-MMP at either the T567^43^ or S577 residues within the cytoplasmic tail, which may, in turn, regulate Y573 phosphorylation. Alternatively, NIK may regulate MT1-MMP at the step of furin cleavage.^19, 20^ Collectively, this study is the first to demonstrate that NIK expression promotes tumor cell invasion by regulating MT1-MMP at the post-transcriptional level.

We demonstrate a strong correlation between cell invasion and expression of NIK at the protein and mRNA levels (Figure 1, Supplementary Figure 1). This correlation is consistent with analyses of both low- and high-grade glioma databases, indicating that increased expression of NIK and MT1-MMP is associated with poor patient survival (Figure 7). Therefore, high NIK and MT1-MMP co-expression may be key prognostic indicators, not only for high-grade gliomas, but also for LGGs that are likely to progress to more aggressive, therapy-resistant tumors. In support of this hypothesis, NIK was recently identified in an analysis of protein interaction networks associated with glioma chemoresistance.^44^

Our study is the first to link expression of NIK to pro-invasive cell shape phenotypes (Figure 2), which have a central role in driving cancer cell dissemination through healthy tissue.^45^ NIK expression significantly enhances pMT1-MMP localization within pseudopodia, to promote increased invasion of 3D matrices (Figures 3,4 and 5 and Supplementary Figure 8). The expansion of pseudopodia length in 2D coupled with increased invasion responses in 3D matrices suggest that NIK may enhance or stimulate the transition from invadopodia to pseudopodia,^46, 47^ which is critical for cell invasion.^45^ Interestingly, differences in cell shape were recently shown to influence the extent of canonical NF-κB activation.^48^ Although NIK does not require RelA/p65 to promote cell invasion and localization of pMT1-MMP to pseudopodia (Figure 5), NIK may be part of a feedback mechanism that tunes the levels of canonical NF-κB activation through regulation of cell shape.

Current therapeutic strategies for targeting NF-κB signaling in high-grade glioma, as well as other tumors, focus on inhibition of the canonical/p65-mediated pathway,^49^ which has well-established tumor-promoting functions.^50, 51^ Here, we have established new roles for NIK in regulating MT1-MMP activity and tumor cell invasion that are independent of RelA/p65, underscoring the importance of developing treatment strategies that target both the canonical and noncanonical NF-κB pathways. Indeed, because invadopodia and pseudopodia formation drive dissemination and metastasis of several cancers,^45^ inhibition of NIK may be an efficacious therapeutic approach in many invasive tumor types.

## Materials and methods

### Reagents

Collagen type I was isolated and prepared as previously described^52^ or purchased from Corning, Corning, NY, USA (#354249). rhTNFα (#G5241) was obtained from Promega, Madison, WI, USA; rhTWEAK (#310-06) was purchased from PeproTech, Rocky Hill, NJ, USA; TAPI-2 was obtained from Calbiochem, San Diego, CA, USA. MG132 was purchased from Cell Signaling Technology, Danvers, MA, USA (#2194S). Anti-cortactin was purchased from Upstate, Lake Placid, NY, USA (# 05-180); anti-p65 (SC-8008) and anti-RelB (SC-226) were from Santa Cruz Biotechnology, Dallas, TX, USA; anti-pMT1-MMP-Y573 was custom-generated from 21st Century Biochemicals, Marlboro, MA, USA,^21, 53, 54^ anti-MT1-MMP was from EMD Millipore, Billerica, MA, USA (MAB3328); anti-α-Actin was from Calbiochem (CP01); AF488 Phalloidin (A12379) and AF-647 Phalloidin (A22287) were purchased from ThermoFisher Scientific/Life Technologies/Invitrogen (Grand Island, NY, USA), anti-NFKB2/p100/p52 (CST4882), p-p65-Ser536 (CST3033) and NIK (#4994, for immunoblot) were purchased from Cell Signaling; anti-GAPDH (ab8245) and anti-NIK (ab7204, for immunofluorescence) were purchased from Abcam, Cambridge, MA, USA. DAPI was purchased from Invitrogen (#D1306).

### Cells

BT25 and BT114 glioma cell lines were described previously^55^ and maintained as tumorspheres in neural stem cell medium (Dulbecco's modified Eagle's medium/F12, 1 × B-27 Supplement minus Vitamin A, 1 × Glutamax, 50 ng/ml EGF, 50 ng/ml bFGF, 1 × Pen/Strep). Spontaneously immortalized MEFs from *p65*^*−/−*^*, cRel*^*−/−*^*p65*^*−/−*^ animals and their corresponding WT MEFs were a gift from Dr Albert S. Baldwin Jr (UNC, Chapel Hill, NC, USA); *RelB*^*+/+*^ and *RelB*^*−/−*^ MEFs were a gift from Dr Denis Guttridge (The Ohio State University), and *NIK*^*+/+*^ and *NIK*^*−/−*^ MEFs were a gift from Dr Robert Schrieber (University of Washington). HEK293FT cells were purchased from Invitrogen. MEF and HEK293FT cells were cultured in Dulbecco's modified Eagle's medium+10% fetal bovine serum (Invitrogen). All cell lines were free of mycoplasma contamination and were not re-authenticated.

### Constructs

pLenti6 overexpression constructs for NIK were generated by subcloning cDNA into pLenti6-V5-DEST (Addgene, Cambridge, MA, USA) using the GATEWAY Cloning System. Luciferase (Promega, Madison, WI, USA) coding sequences were subcloned into pLenti6-V5-DEST and used as controls for NIK overexpression. NIK(S867A) mutation was obtained by PCR cloning using oligo primers containing the mutation.

### CRISPR-Cas9 *NIK* gene knockout cell clones

Lenti-CrispR-v2 (Addgene) was used to express both *Cas9* and human *NIK* sgRNA (sgNIK-1: GCUCCUUCGGAGAGGUGCAC, sgNIK-2: GAAAGCGUCGCAGCAAAGCC or sgNIK-3: AGUACCGAGAAGAAGUCCAC). BT25 cells were transduced with mixture of lentiviruses carrying these sgRNAs and then selected against 0.6 μg/ml puromycin; 1507 bp and 33 bp deletions around sgRNA target sequences were identified by sequencing. Loss of NIK expression was confirmed by immunoblot, qPCR and/or immunofluorescence microscopy analysis of puromycin-resistant cells. Single colony cells were isolated by serial dilution and experiments were repeated with at least two clones.

### Lentiviral production

HEK293FT cells were transfected with 24 μg of lentiviral plasmids using 72 μg of polyethyleneimine (Polysciences Inc., Warrington, PA, USA). Lentiviruses were harvested after 3 days and used to infect 2 × 10^5^ BT114 or MEF cells. Transduced cells were grown for 72 h in neural stem cell medium or Dulbecco's modified Eagle's medium with 10% fetal bovine serum containing 6 μg/ml or 0.1 μg/ml blasticidin (Invivogen, San Diego, CA, USA), respectively, to select cells with stable transduction.

### Invasion assays

Invasion assays were performed as previously described;^52^ 3D collagen type I matrices were prepared at a final concentration of 2 mg/ml, containing indicated concentrations of TWEAK (0 or 10 ng/ml). Following collagen polymerization, dissociated cells (40 000 cells/100 μl Dulbecco's modified Eagle's medium) were seeded and incubated at 37 °C with 5% CO_2_. To inhibit MMPs, DMSO, 2.5 or 5 μM TAPI-2 was preincubated with the cells for 20 min at 37 °C before seeding onto collagen matrices. After 24–48 h, invading cells were fixed with 3% glutaraldehyde in phosphate-buffered saline and stained with 0.1% toluidine blue/30% methanol. Results from at least three independent experiments are shown.

### Quantification of invasion responses

Invasion density was quantified manually in toluidine blue-stained invasion assays as the average number of structures invading past the monolayer, per field, using an eyepiece fit with a 10 × 10 ocular grid. At least four wells per treatment group were counted, per experiment, for three independent experiments. After fixation and staining, collagen gels were removed from the well, cut and imaged from the side using an Olympus CKX41 microscope (Walthan, MA, USA) with a Q-Color 3 camera or a Nikon Ti Eclipse inverted microscope (Tokyo, Japan) and DS-Fi1 5-Meg Color C-Mount Camera at × 20 magnification.

### RNA isolation, cDNA synthesis and quantitative RT–PCR

Total RNA was isolated from cells using PurelinkTM RNA Mini Kit (Life Technologies). cDNA was synthesized from 1 μg of total RNA using Super-Script III Reverse Transcriptase (Life Technologies) following the manufacturer's instructions. Quantitative RT–PCR was performed using SYBR Green PCR Master Mix (Applied Biosystems, Foster City, CA, USA). Expression of mRNA was normalized to either GAPDH or RPLP0 expression levels. The following primers were used: GAPDH 5′-AATGAAGGGGTCATTGATGG-3′, 5′-AAGGTGAAGGTCGGAGTCAA-3′ RPLP0 5′-TCGTCTTTAAACCCCTGCGTG-3′, 5′-TGTCTGCTCCCACAATGAAAC-3′ MMP14 (human) 5′-TGCCTACCGACAAGATTGATG-3′, 5′-ATCCCTTCCCAGACTTTGATG-3′ MMP14 (mouse) 5′-GGATGGACACAGAGAACTTCG-3′, 5′-TTTTGGGCTTATCTGGGACAG-3′ NIK 5′-TTCAGCCCCACCTTTTCAG-3′, 5′-ACGCTTTCCCTTCCAACAC-3′. Three independent experiments were performed in triplicate wells. qRT–PCR data were analyzed using StepOne Software (version 2.1). ΔΔCT values were normalized to GAPDH expression values for each sample, set relative to indicated treatment and converted to 2^*−*ΔΔCT^ to compare the relative mRNA fold-change expression between treatment groups.

### Immunostaining and imaging

Cells (2.5 × 10^5^) were seeded on collagen (50 μg/ml)-coated 12-mm coverslips in a 24-well plate. After 16 h incubation, cells were treated as indicated, fixed in 4% paraformaldehyde, permeabilized with 0.5% Triton X-100 in phosphate-buffered saline and blocked overnight in 0.1% Triton X-100, 1% BSA/1% goat serum in TBS at 4 °C. To examine the effects of TWEAK treatment on pMT1-MMP localization in MEFs, cells were serum-starved for 2 h, treated with TWEAK (10 ng/ml) for 4 h and subsequently fixed and processed as described above. DAPI (1.09 μM; Invitrogen) was used for staining nuclei. Cells were imaged using a Nikon TI A1R inverted confocal microscope to capture Z-stacked images with a 0.225 μm step size. Fluorescence was quantified using Nikon Elements Software (see Supplementary Figure 3A).

### MT1-MMP activity assays

MT1-MMP activity assays were performed on cell lysates (not conditioned media) using a Sensolyte, AS-72025 kit, with the following modifications; to inhibit soluble MMPs and specifically evaluate MT1-MMP activity after 8–24 h invasion, lysates from cells invading collagen were incubated with conditioned media from TIMP-1-transfected HEK293FT cells.^54^ The MT1-MMP-sensitive substrate was added to equal amounts of cell lysate and incubated for 30 min. Fluorescence intensity was measured at Ex/Em 490/520 nm using Victor X3 Multilabel Reader (PerkinElmer, Waltham, MA, USA). Relative fluorescence units were calculated by subtracting the control reading from each sample and averaging the remaining relative fluorescence unit value of the replicates. Gelatin zymography was performed as previously described.^56^

### Western/immunoblotting

Whole-cell extracts were prepared using RIPA. To detect NIK protein expression, cells were pre-treated with MG132 (5 μM) ±TWEAK (10 ng/ml), as indicated. Proteins were subjected to western blotting as previously described.^3, 54^

### Immunohistochemical analysis of mouse xenograft tumors

Intracranial xenografts were previously performed.^3^ Sections (10 μm) from OCT-embedded tumors were immunostained with incubated with NIK-specific antibody (ab7204) and Alexa Fluor 488-secondary antibody and DAPI (Invitrogen). Images were acquired using a Nikon TI A1R inverted confocal microscope. Adjacent tumor sections were used for immunohistochemical detection of pMT1-MMP (Y573) using the Vectastain ABC elite kit (Vector Laboratories, Burlingame, CA, USA) and DAB peroxidase substrate (Vector Laboratories) with hematoxylin counterstain. Images were acquired with a Nikon Eclipse Ti microscope.

### *Ex vivo* glioma tumor tissue

The use of de-identified human GBM tissue was approved by the Institutional Review Boards (IRBs) of Saint Joseph Regional Health Center, Bryan, TX, USA (IRB2012-001) and Texas A&M University (IRB2014-0318D), and informed consent was obtained from all subjects. Sections of human glioma biopsy samples were stained with DAPI and antibodies specific for pMT1-MMP (Y573) and NIK (ab7204, which was pre-conjugated to AF488 using an antibody labeling kit (Molecular Probes/Invitrogen, A20181)). Immunostaining was performed sequentially using the pre-labeled NIK antibody last. Imaging was performed as described above.

### Glioma database analysis

Gene expression data for GBM patients from The Cancer Genome Atlas (TCGA), Cell 2013 data set^57^ and LGG patients from the TCGA (Provisional raw data set, NCI) were downloaded from cBioPortal for Cancer Genomics^37, 38^ and analyzed using Prism 6 (Graphpad Software, Inc., La Jolla, CA, USA). Kaplan–Meier plots were generated from GBM and LGG patients with increased NIK (MAP3K14) and MT1-MMP (MMP14) mRNA expression with a z-score of 1.5. GBM data set can be accessed at http://bit.ly/1IFXIKA and LGG data set can be accessed at http://bit.ly/1IFY43S.

### Statistical analyses

All statistical analyses were performed using Prism 6 (Graphpad Software, Inc.). For all experiments, at least three independent experiments were performed with *n*⩾3 replicate samples per experiment. No statistical method was used to predetermine sample size. Experiments were not randomized. Investigators were not blinded to data allocation (with the exception of assessing cell size and pseudopodial length in Figures 2c–f, Supplementary Figure 3A). Unpaired Student's *t*-test was performed on data comparing two groups, assuming similar variance. One-way or two-way analysis of variance with Holm-Sidak or Tukey's *post hoc* test for multiple comparisons and multiplicity-adjusted *P*-values are reported.^58^ Kruskal–Wallis with Dunn's post test was used to assess statistical significance between log2-base NIK and MT1-MMP gene expression data. Survival and disease-free patient data were analyzed using a Log-rank Mantel-Cox test. Unpaired Welch *t*-test was used to analyze mean survival GBM patients. In all studies, *P*-values <0.05 were considered significant.
