# Supplementary material for: NIK regulates MT1-MMP activity and promotes glioma cell invasion independently of the canonical NF-κB pathway
Source: Oncogenesis. 2016 Jun 6;5(6):e231–. doi: 10.1038/oncsis.2016.39 (PMC4945740; doi:10.1038/oncsis.2016.39)
Supplement: Supplementary Figures [file oncsis201639x1.pdf]

Supplemental Figure 1

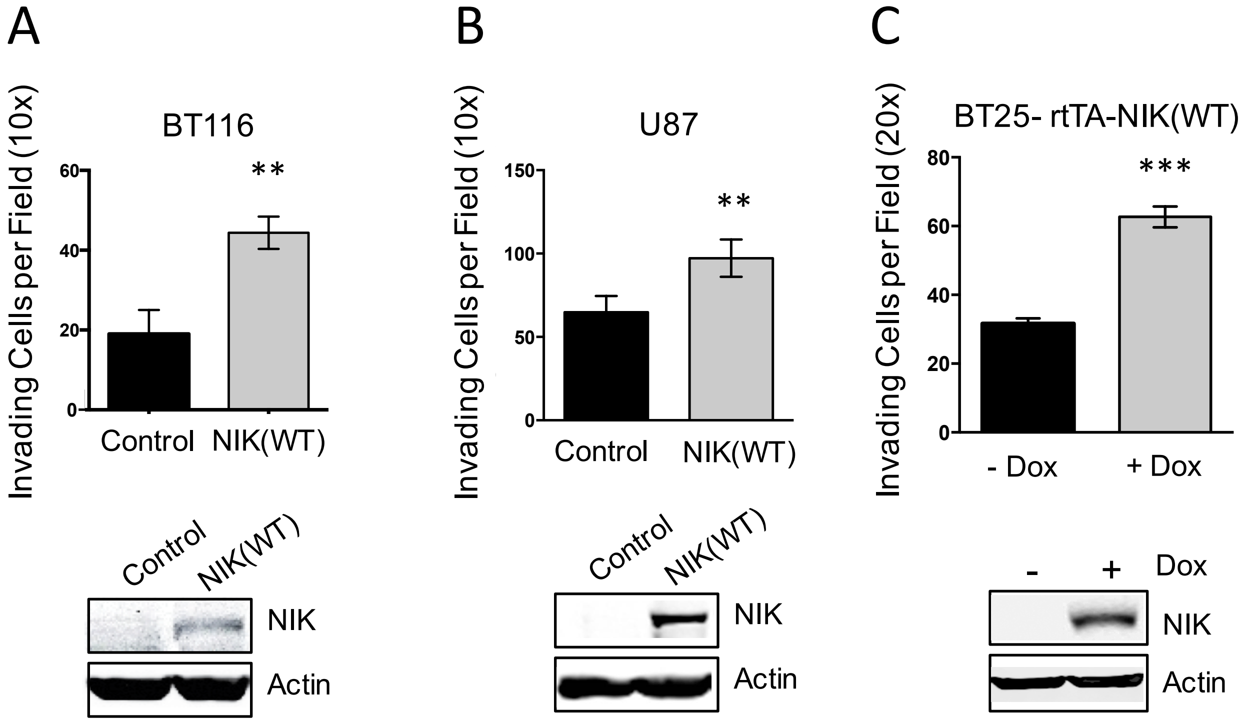

Supplemental Figure 2

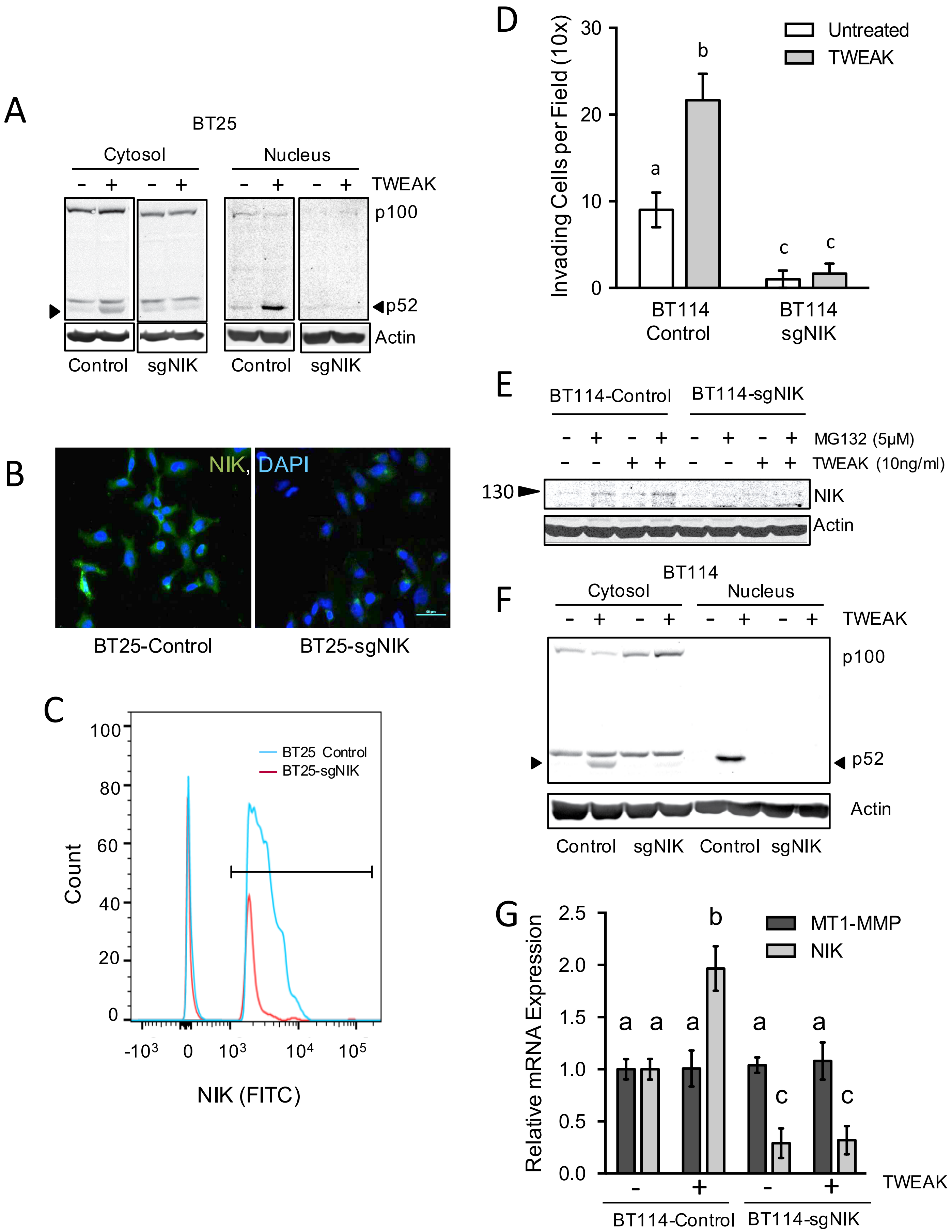

Supplemental Figure 3

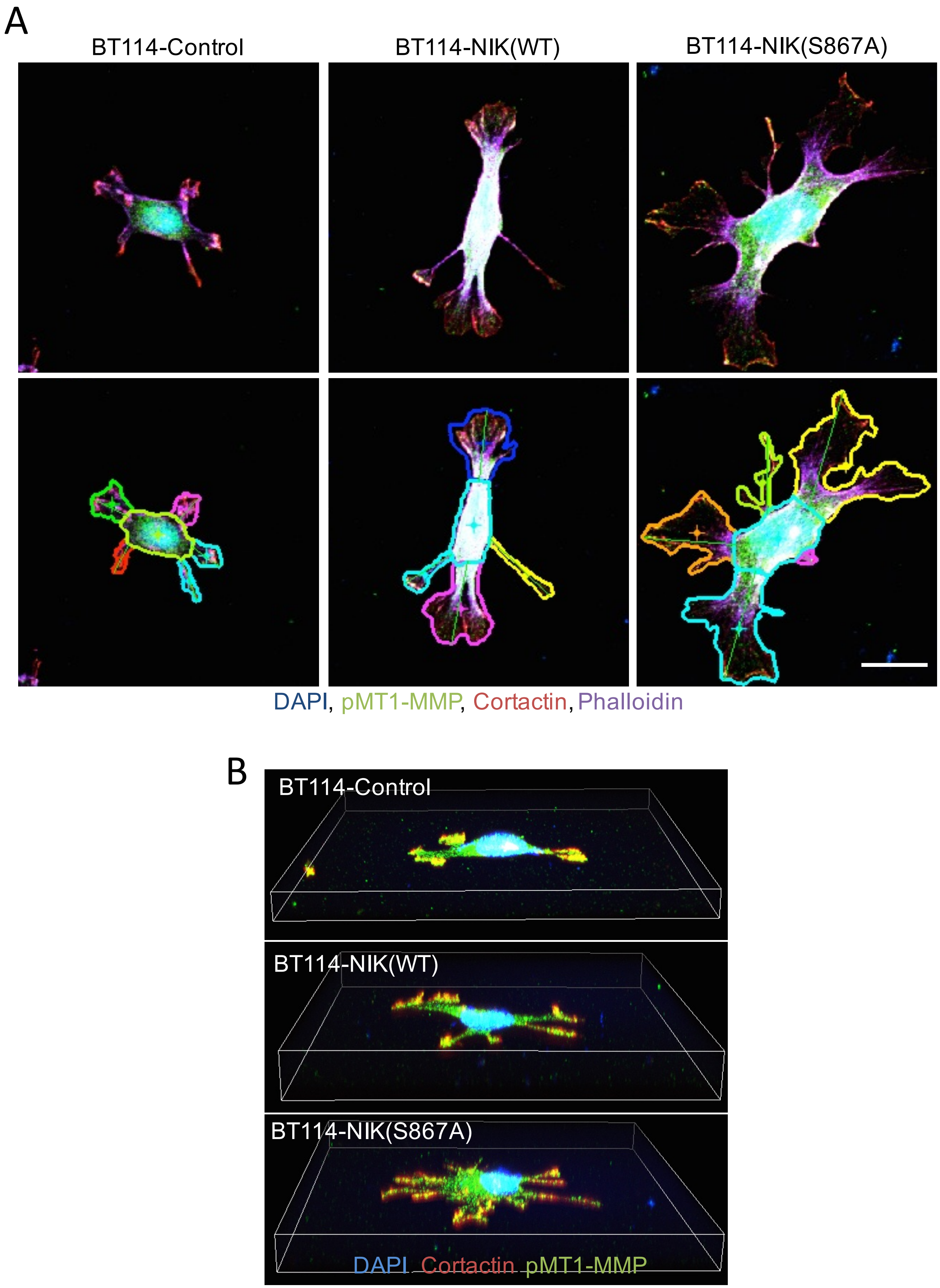

Supplemental Figure 4

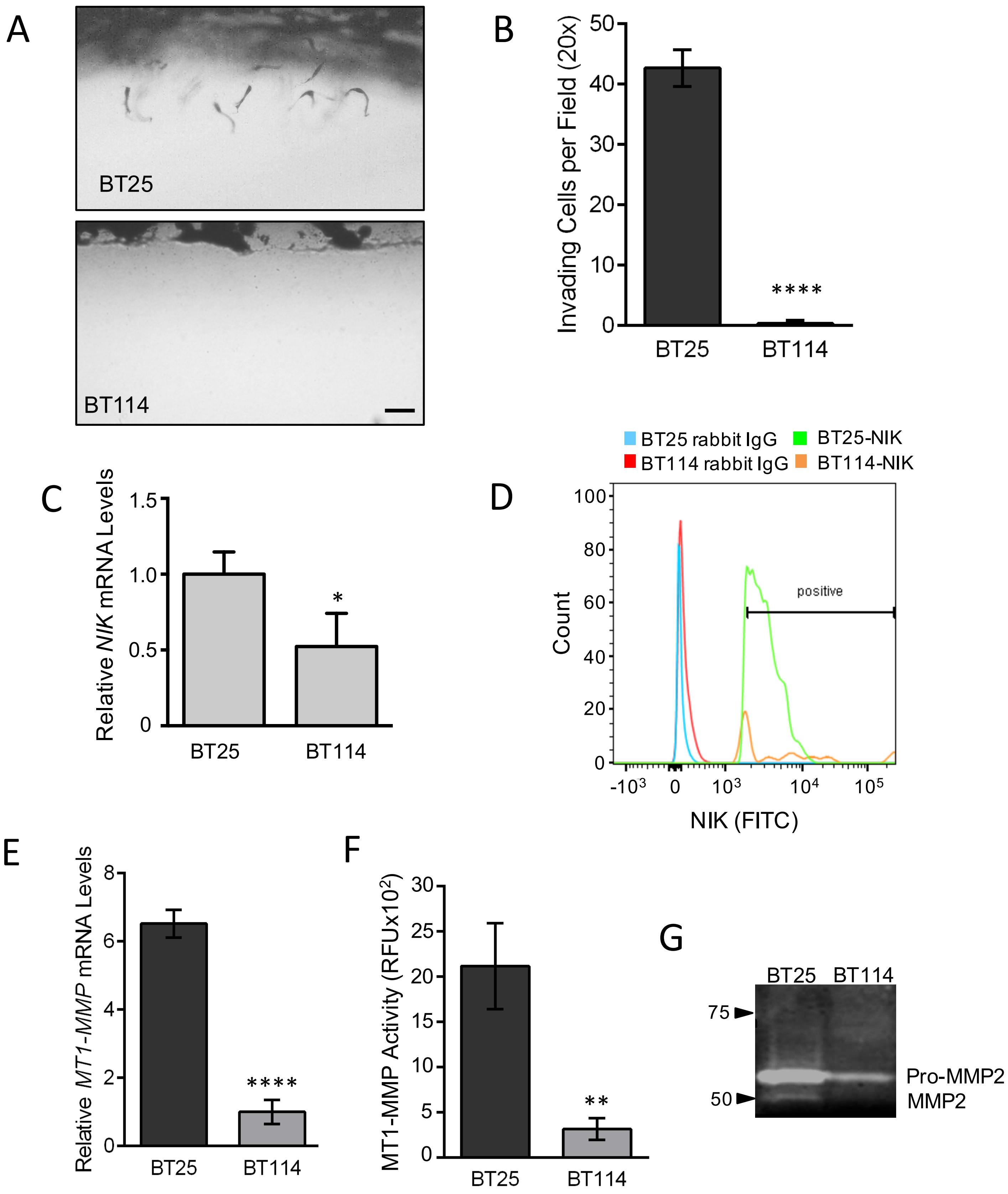

Supplemental Figure 5

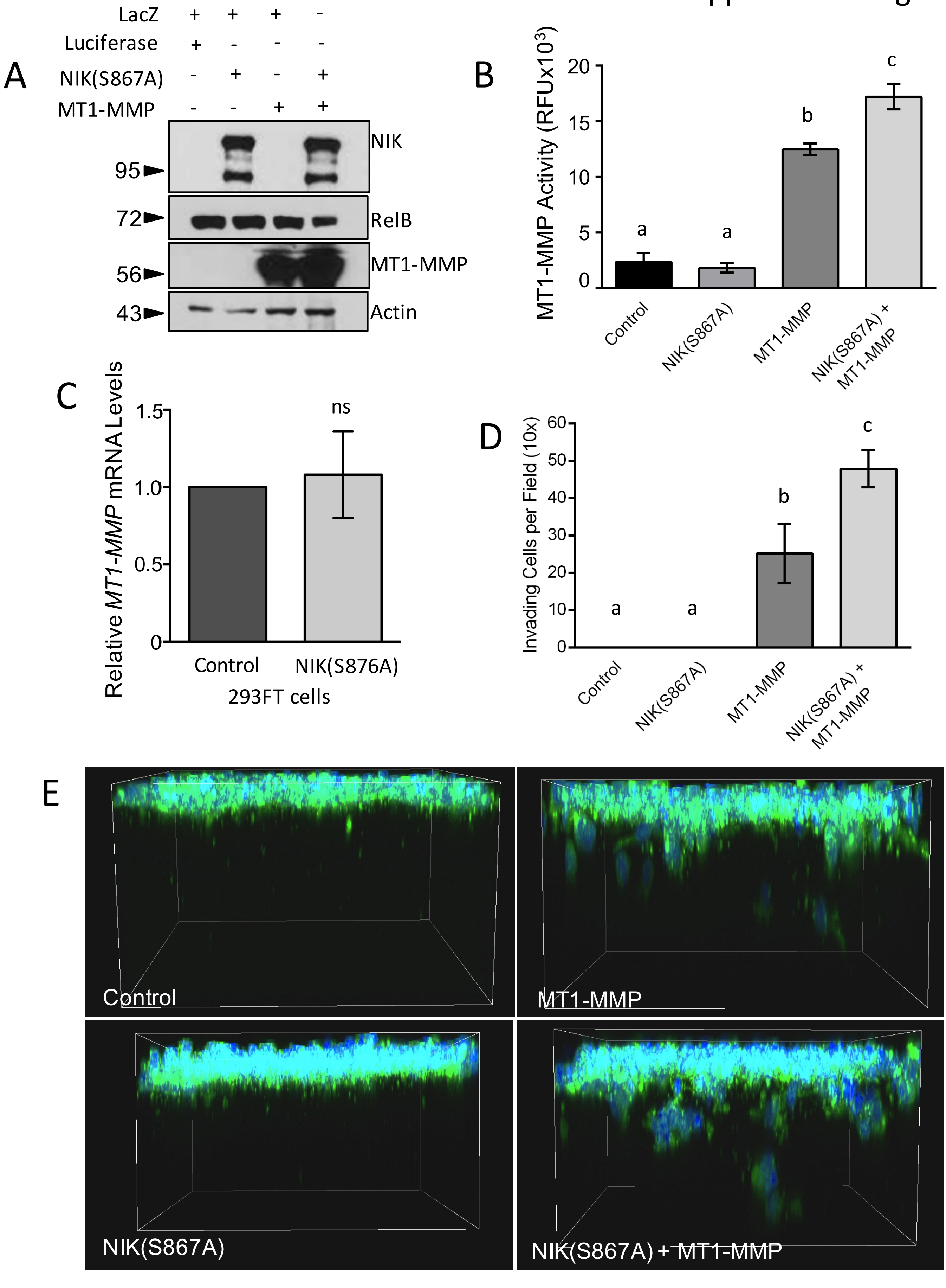

Supplemental Figure 6

A

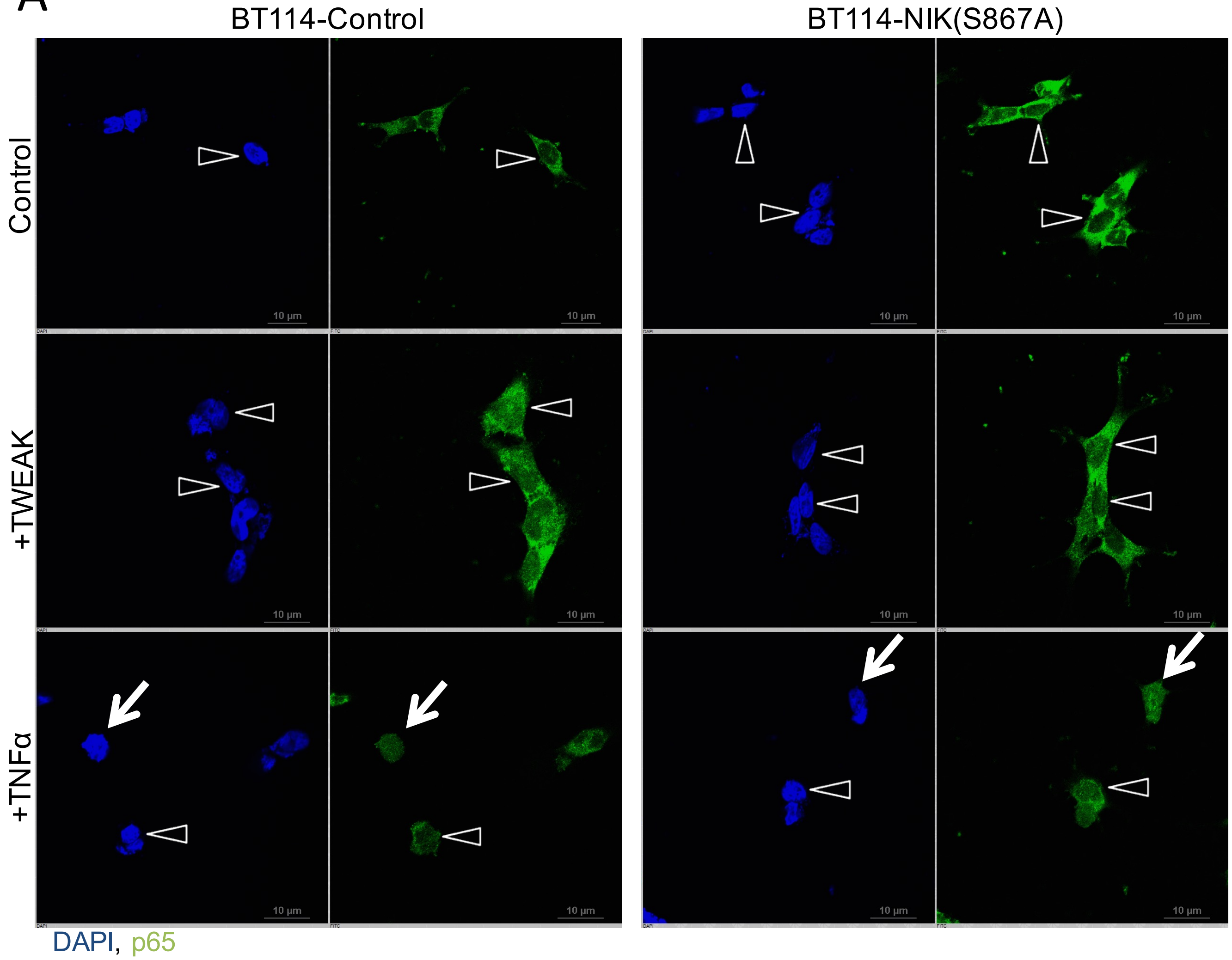

B

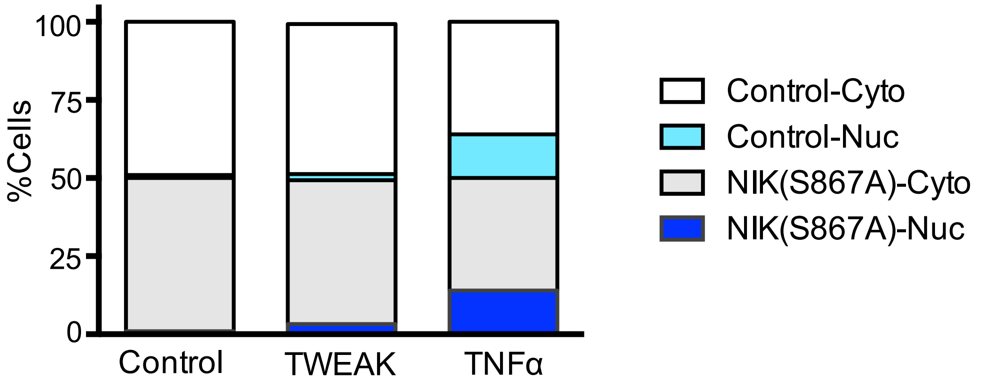

Supplemental Figure 7

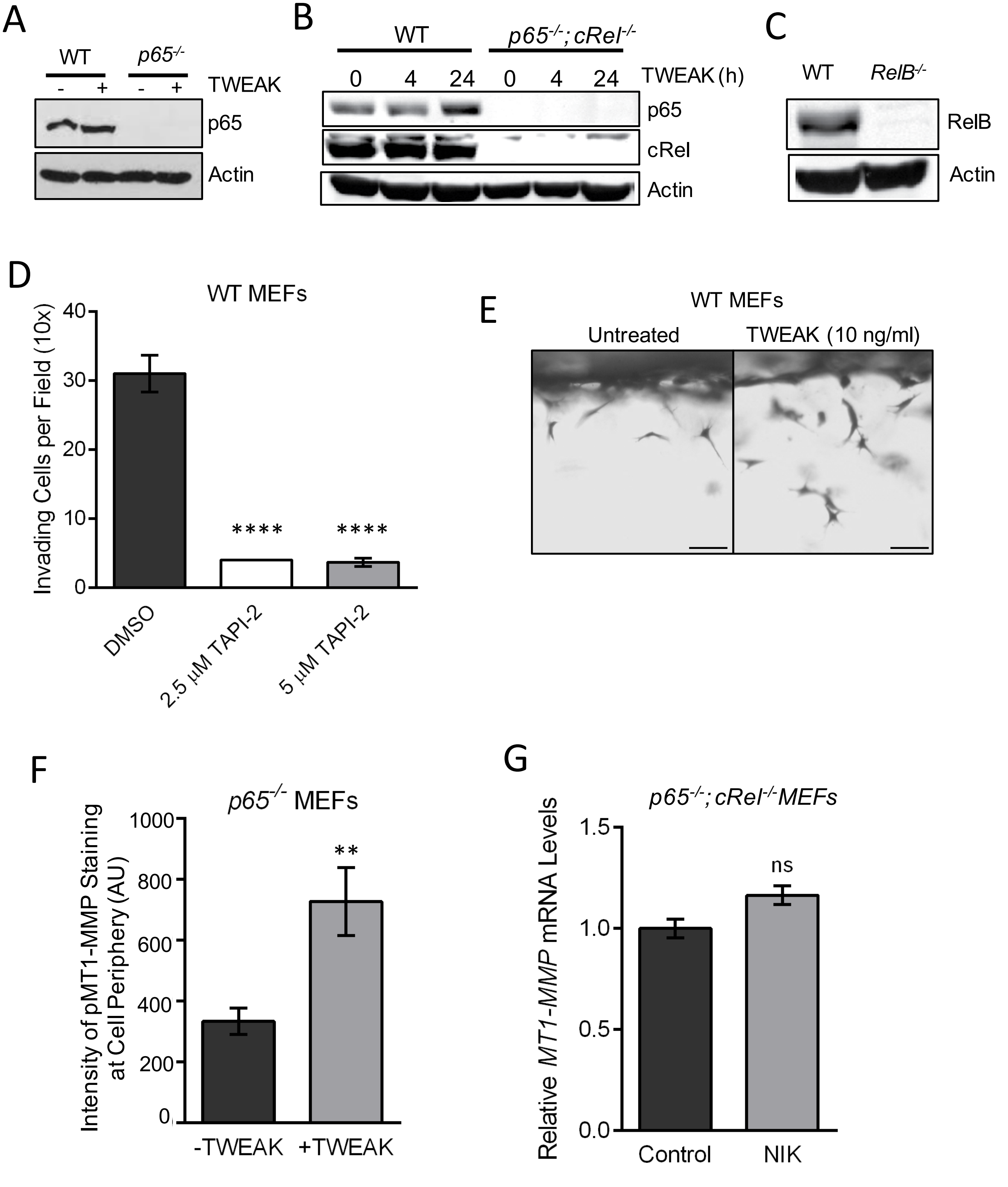

Supplemental Figure 8

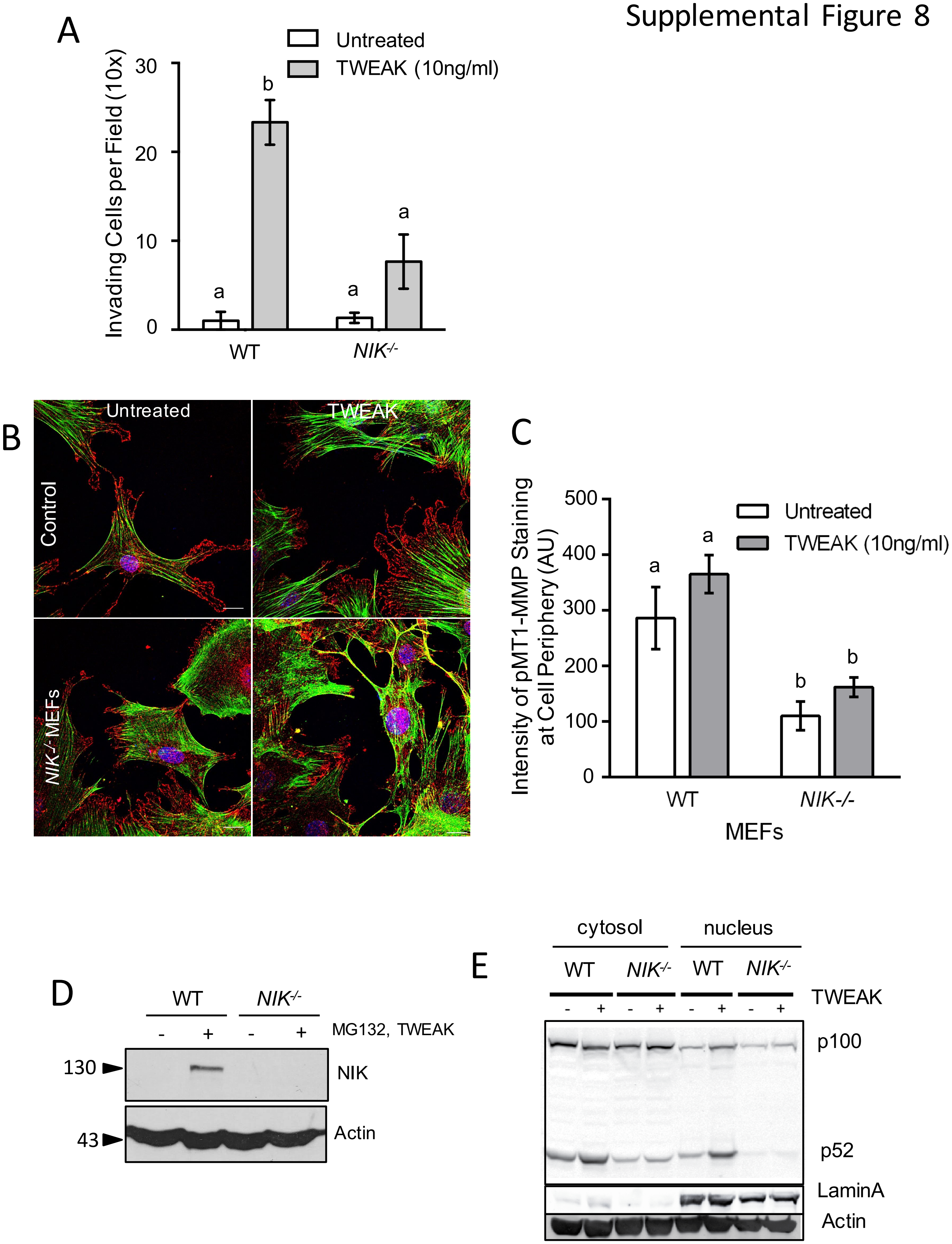

SUPPLEMENTAL FIGURE LEGENDS:

Supplemental Figure 1. NIK promotes invasion in different glioma lines. Supports Figure 1. (A) BT116 cells expressing vector (Control) or NIK(WT) were seeded onto 3D collagen matrices and allowed to invade 24 hours. Graph shows average number of invading cells +/- SD in a 0.25 mm^2 field. At least 4 wells were quantified per cell type for three independent experiments. Unpaired t-test P-value: ** 0.0037 Control vs. NIK(WT). Western blot analysis of BT116 cells expressing vector (Control) or NIK(WT) confirming exogenous NIK expression. **(B)** U87 cells expressing vector (Control) or NIK(WT) were seeded onto 3D collagen matrices and allowed to invade 24 hours. Graph shows average invasion density. At least 4 wells were quantified per cell type for three independent experiments. Unpaired t-test P-value: ** 0.0012 Control vs. NIK(WT). Western blot analysis of U87 cells expressing vector (Control) or NIK(WT) confirming exogenous NIK expression. **(C)** BT25 cells expressing NIK under control of reverse tetracycline-controlled transactivator (rtTA), inducible through doxycycline, were not treated (-Dox) or treated with 5ng/ml (+Dox) for 48 hours before seeding onto collagen matrices and allowing to invade 48 hours. Graph shows average invasion density. At least 4 wells were quantified per cell type for three independent experiments. Unpaired t-test: ***P <0.0001 -Dox vs. +Dox. Western blot analysis of BT25 cells treated with (+Dox) or without doxycycline (-Dox) confirming induced NIK expression.

Supplemental Figure 2. Glioma cells expressing sgNIK have decreased NIK expression. Supports Figure 1. (A) Nuclear and cytoplasmic fractions were isolated from BT25 cells

expressing vector (Control) or sgNIK treated with (+) or without (-) 10 ng/ml TWEAK for 24 hours. Extracts were probed with antibodies directed to Actin and p100/p52 (Cell Signaling Technology, 3017S) to assess p100 processing and p52 nuclear translocation downstream of NIK. **(B)** BT25-Control and BT25-sgNIK cells were seeded onto collagen coated coverslips and immunostained with antibodies directed to NIK (Abcam, ab7204) (green) and DAPI (blue) to demonstrate loss of NIK expression in sgNIK cells, and specificity of antibody. Scale bar = 10 µm. **(C)** Flow cytometry was performed to quantify NIK staining in BT25-Control and BT25-sgNIK cells. $3x10^5$ cells were fixed, permeabilized in 0.3% Triton in PBS, and incubated with antibodies directed to NIK (Abcam, 7204, 1:50 dilution) or isotype controls followed by incubation with FITC-conjugated secondary antibody (Alexa Fluor-488. 1:200). At least 10000 events were analyzed with a BD LSRFortessa™ X-20 cell analyzer and data was analyzed, normalizing to mode, with FLowJo software (v-X). **(D)** BT114-Control and BT114-sgNIK cells were seeded onto collagen matrices with or without 10 ng/ml TWEAK and allowed to invade 24 hours. Representative graph from three independent experiments shows average invasion density counted from three wells per treatment. Statistical analysis was calculated using 2-way ANOVA and Tukey's HSD post test. Different letters indicate statistically significant differences with the following multiplicity adjusted P-values: p=0.0002 for "a vs. b"; p<0.01 for "b vs. c" and "a vs. c". **(E)** Western blot analysis of BT114 cells expressing vector (Control) or sgNIK treated with or without MG132 (5 µM) for 1 hour to prevent NIK degradation and then untreated, or treated with TWEAK (10 ng/ml), as indicated for 5 hours. Whole cell lysates were probed with indicated antibodies. **(F)** Western blot analysis of BT114 cells expressing vector (Control) or sgNIK treated as in Supplemental 2A. Extracts were probed with antibodies directed to Actin and p100/p52 to

demonstrate inhibition of p100 processing and p52 nuclear translocation in BT114-sgNIK cells. **(G)** Quantitative RT-PCR analysis was performed to analyze expression of *MT1-MMP* and *NIK* in BT114-Control and BT114-sgNIK cell untreated or treated with TWEAK (10ng/ml) for 24 hours. Graph shows fold-change *MT1-MMP* and *NIK* expression relative to untreated BT114-Control cells from triplicate wells from one representative experiment that was repeated three times. *RPLP0* expression was used as endogenous control. Statistical analysis of gene expression data was calculated using 2-way ANOVA with Tukey's HSD post test. Different letters indicate statistically significant differences with multiplicity adjusted P-values: a vs. b and b vs. c < 0.0001; a vs. c = 0.0004.

Supplemental Figure 3. Quantification of BT114 pseudopodia. Supports Figure 3. (A) BT114 cells transduced to express vector (Control), NIK(WT), or NIK(S867A) were seeded onto collagen-coated coverslips and stained with DAPI (blue) and antibodies directed to pMT1-MMP (green), cortactin (red), and Alexa Fluor 647-phalloidin (Cy-5). Cells were imaged using confocal microscopy and analyzed using Nikon NIS Elements Software. Z-stacks were compressed and analyzed. Only single cells containing one nucleus and not in contact with another cell were used for quantifications. The average pseudopodial length was then quantified by drawing a line starting from the line tangent to the cell body, and extending to the furthest cortactin-pMT1-MMP- positive point of the protrusion. The average cell area was quantified by creating an ROI outlining the cell. At least 65 protrusions per treatment group were used for blinded quantifications. Scale bar=10 µm. **(B)** Immunofluorescence staining of BT114 cells expressing

Control, NIK(WT) or NIK(S867A) grown on collagen-coated coverslips was performed to demonstrate colocalization between pMT1-MMP and cortactin at pseudopodial structures. Cells were stained with DAPI (blue), pMT1-MMP (green), and cortactin (red), and imaged using confocal microscopy. Volumetric views of confocal Z-stacks were made using Nikon NIS Elements software and rotated to show 3D view of pseudopodia.

Supplemental Figure 4. Glioma cell invasiveness correlates with MT1-MMP expression and activity. Supports Figure 3. (A) BT25 and BT114 glioma cell lines were seeded onto collagen matrices and allowed to invade 24 hours. Cultures were fixed in 3% glutaraldehyde, stained with toluidine blue, and imaged from the side to visualize invasion responses. Scale bar = 50 µm. **(B)** Invasion density was quantified as the average number of invading cells $\pm$SD in a 0.25 mm^2 field. At least 4 wells were quantified per cell type for three independent experiments. Unpaired Student's test P-value ****p<0.0001. **(C)** Quantitative RT-PCR was performed to analyze *NIK* mRNA expression in BT25 and BT114 glioma cells. Graph shows fold change *NIK* expression relative to BT25 cells, averaged from triplicate wells from one representative experiment. Unpaired test P-values:*p=0.0351. **(D)** NIK protein expression in BT25 and BT114 cells was compared using flow cytometry with antibodies directed to NIK (Abcam, 7204) or isotype controls, as described for Supplemental Figure 2C. **(E)** Quantitative RT-PCR was performed to analyze expression of *MT1-MMP* in BT25 and BT114 cells. Graph shows fold-change *MT1-MMP* expression relative to BT114 cells, averaged from triplicate wells from one representative experiment. Student's t-test P-value ****p<0.0001. **(F)** MT1-MMP activity was

assayed using the Sensolyte 520-MMP-14 Assay kit (AnaSpec) with extracts made from the indicated cells after 8 hours of invasion and incubation with TIMP-1 conditioned media. Graph from one representative experiment (n=3) is depicted, showing average RFU (relative fluorescence units) from three replicates per cell type. Unpaired Student's t-test P-value **p=0.0031. **(G)** Gelatin zymogram was performed to evaluate matrix degradation by BT25 and BT114 cells, as previously described [1].

Supplemental Figure 5. NIK increases MT1-MMP activity and invasion in a heterologous HEK293FT system. Supports Figure 4. HEK293FT cells were transfected to express LacZ and Luciferase, LacZ and NIK(S867A), LacZ and MT1-MMP, or NIK(S867A) and MT1-MMP. Cells were seeded onto 1.5 mg/ml collagen matrices containing 1 ⍰M LPA and allowed to invade 72 hours. **(A)** Whole cell lysates were probed with antibodies directed against NIK, RelB, MT1-MMP and Actin using western blotting. **(B)** MT1-MMP activity assays were performed as described in Figure 3E, using lysates made from HEK293FT cells after 24 hours of invasion. RFU= relative fluorescence units. One-way ANOVA with Tukey's HSD post test was used for statistical analysis. Different letters indicate statistical significance. Multiplicity adjusted P-value <0.0001 for all comparisons. **(C)** Quantitative RT-PCR was performed to analyze *MT1-MMP* mRNA expression in 293FT cells transfected to express luciferase (Control) or NIK(S867A). Graph shows fold change *MT1-MMP* expression relative to 293FT cells expressing Luciferase (Control), averaged from triplicate wells from one representative experiment. Unpaired Student's t-test P-values = 0.6453 (ns). **(D)** Quantification of average number of invading cells per field ± SD. At least 4

wells were quantified per cell type for three independent experiments. A representative graph is shown. Significance was calculated using one-way ANOVA with Tukey's HSD test and letters indicate statistically significant differences. Multiplicity adjusted P-values: $p < 0.0001$ for all comparisons. **(E)** Invasion assays were fixed with 4% paraformaldehyde, stained with Alexa Fluor 488-phalloidin (green) and DAPI (blue) and imaged using confocal microscopy. Z-stacks were compressed and rotated to demonstrate side-view of invasion.

Supplemental Figure 6. NIK expression or TWEAK treatment does not induce canonical NF-κB. Supports Figure 5. BT114 cells expressing Vector (Control) or NIK(S867A) seeded onto collagen-coated coverslips were treated with nothing (Control), 10 ng/ml TWEAK for 4 hours or 20 ng/ml TNFα for 30 minutes, as a control to demonstrate p65 movement to nucleus, before fixing with 4% paraformaldehyde. **(A)** Coverslips were immunostained with antisera directed to p65 (green), stained with DAPI (blue) to visualize nuclei, and imaged using confocal microscopy. Arrowheads indicate p65-negative nuclei. Arrows indicate p65-positive nuclei. Scale bars = 10 µm **(B)** 50 cells per treatment per experiment (n=3) were examined for the percentage of p65 localization to the nucleus (Nuc) or cytoplasm (Cyto). A representative experiment is shown.

Supplemental Figure 7. MEF invasion is MMP dependent and MT1-MMP expression and localization to pseudopodia increases with TWEAK treatment. Supports Figure 5. (A) Western blot analysis confirms loss of p65 in null MEFs ± TWEAK treatment (10ng/ml for 24 hours) using indicated antibodies. **(B)** WT and *p65*$^{-/-}$*; cRel*$^{-/-}$ MEFs were treated with 10 ng/ml TWEAK for

indicated times. Western blot analysis confirms loss of NF-κB proteins, p65 and cRel in null MEFs, using indicated antibodies (cRel: Cell Signaling Technology, CST4727). **(C)** Western blot analysis confirms loss of RelB in null MEFs, using indicated antibodies. **(D)** Average invasion density quantification ± SD using WT MEFs treated with vehicle control (DMSO) or the MMP inhibitor TAPI-2 (2.5 or 5 µM) before seeding onto 3D collagen matrices. Four wells were quantified per experiment for three independent experiments. Graph shows a representative experiment. Statistical significance was calculated with one way ANOVA with Dunnett's multiple comparisons test where ****p<0.0001 for 2.5 and 5 µM TAPI-2 vs. DMSO. **(E)** Untreated or TWEAK-treated WT MEFs were seeded onto collagen matrices and allowed to invade 24 hours. Side view images of invasion are shown. Scale bar = 50 µm. **(F)** Blinded quantification of pMT1-MMP intensity beyond actin-rich protrusions expressed in arbitrary units (AU) ± SEM of images from Figure 5B of $p65^{-/-}$ MEFs ± TWEAK (10ng/ml). At least 30 cells per treatment from three independent experiments were used for quantifications. **p<0.01 using unpaired Student's *t*-test. **(G)** Quantitative RT-PCR was performed to analyze MT1-MMP expression in $p65^{-/-}$;$cRel^{-/-}$ MEFs expressing vector alone (Control) or NIK. *GAPDH* expression was used as endogenous control. Gene expression data was averaged from triplicate wells from one representative experiment of three total experiments. Unpaired Student's t-test P-values = 0.0654 (ns).

Supplemental Figure 8. Loss of NIK in MEFs abrogates surface localization of pMT1-MMP in response to TWEAK. Supports Figure 5. (A) Invasion density of WT $NIK^{-/-}$ MEFs, untreated or

treated with TWEAK (10 ng/ml) and allowed to invade 3D collagen matrices for 48 hours. Three wells were quantified per treatment in three independent experiments. Graph depicts one representative experiment. Statistical analysis of invasion data was calculated using 2-way ANOVA. Different letters indicate statistically significant differences with multiplicity adjusted P-value of p<0.0001 for all comparisons. **(B)** WT and *NIK*$^{-/-}$ MEFs were seeded onto collagen coated coverslips, and treated and stained with anti-pMT1-MMP antibody (red), Alexa Fluor 488-phalloidin (green), and DAPI (blue), and imaged using confocal microscopy. Scale bar = 10 µm. **(C)** Intensity of pMT1-MMP staining beyond actin-rich protrusions expressed in arbitrary units (AU) ± SEM of images from Supplemental Figure 8B of WT and *NIK*$^{-/-}$ MEFs untreated or treated with 10ng/ml TWEAK. Statistical significance was calculated using 2-way ANOVA with Tukey's HSD post test. Statistically significant differences are indicated by different letters. Multiplicity adjusted P-values: <0.0001 for "a vs. b." **(D)** WT and *NIK*$^{-/-}$ MEFs were treated with or without MG132 (5 µM) for 1 hour to prevent NIK degradation and then untreated, or treated with TWEAK (10 ng/ml), as indicated for 5 hours. Western blot analysis using indicated antibodies confirms loss of NIK protein in *NIK*$^{-/-}$ MEFs. **(E)** Nuclear and cytoplasmic fractions were isolated from WT and *NIK*$^{-/-}$ MEFs treated ± 10 ng/ml TWEAK for 24 hours. Extracts were probed with antibodies directed to Actin and p100/p52 to evaluate p100 processing and p52 nuclear translocation in *NIK*$^{-/-}$ MEFs. Antibodies directed to LaminA (Cell Signaling Technology, CST4777) were used to confirm isolation of nuclear extracts.

REFERENCES

1. Bayless KJ, Davis GE. Sphingosine-1-phosphate markedly induces matrix metalloproteinase and integrin-dependent human endothelial cell invasion and lumen formation in three-dimensional collagen and fibrin matrices. Biochemical and biophysical research communications. 2003;312(4):903-13.
